# F7 Drives Gastric Cancer Metastasis Through Anoikis Resistance and Tumor Microenvironment Remodeling

**DOI:** 10.1002/advs.77095

**Published:** 2026-08-17

**Authors:** Lei Gao, Qinying Han, Yunpeng Wang, Xuemei Li, Bofang Wang, Baohong Gu, Weidong Li, Lin Xiang, Ying Zhang, Hao Chen

**Affiliations:** ^1^ The Second Hospital & Clinical Medical School Lanzhou University Lanzhou People's Republic of China; ^2^ Cancer Center, Shanghai General Hospital Shanghai Jiao Tong University School of Medicine Shanghai People's Republic of China; ^3^ Department of Laboratory Medicine, the First Medical Centre Chinese PLA General Hospital Beijing People's Republic of China

**Keywords:** extracellular matrix, gastric cancer, immune checkpoint inhibitor, immunosuppression, tumor microenvironment (TME)

## Abstract

Coagulation factor VII (F7) has been implicated in tumor progression; however, its role in gastric cancer metastasis and immune evasion remains incompletely understood. In this study, we identified F7 as a clinically relevant driver of gastric cancer. F7 was highly expressed in tumor tissues and closely associated with lymph node metastasis, poor prognosis, and reduced CD8^+^ T‐cell infiltration. Tumor‐derived autocrine F7 interacted with ITGA2 on the cancer cell surface and activated ITGA2‐associated FAK–NF‐κB signaling and promoted invasion, migration, and anoikis resistance. F7 induced PD‐L1 expression in a manner partly dependent on ITGA2‐mediated PI3K–AKT signaling and promoted collagen I deposition in liver metastatic lesions, which was associated with reduced CD8^+^ T‐cell infiltration. Co‐immunoprecipitation showed that the K1.2 and K1.3 regions of F7 mediated its interaction with the N‐terminal extracellular region of ITGA2. Further investigations identified HNF4A as an upstream transcription factor of F7, with anoikis‐associated stress enhancing HNF4A nuclear accumulation and F7 transcription. Notably, ThonningianinA (THA) was identified as a potential F7‐targeting small molecule that suppressed F7‐associated malignant phenotypes, reduced tumor growth, and enhanced the antitumor efficacy of anti–PD‐1 therapy. These findings support F7 as a potential therapeutic target for metastatic gastric cancer.

## Abstract 2

Tumor‐derived F7 drives gastric cancer metastasis and immune evasion by activating ITGA2‐associated signaling, enhancing PD‐L1 expression, and suppressing CD8^+^ T‐cell infiltration and effector function. Anoikis stress induces HNF4A‐mediated F7 transcription, while pharmacological targeting of F7 with Thonningianin A suppresses gastric cancer metastatic progression and enhances anti–PD‐1 efficacy.

## 1 Introduction

Gastric cancer remains one of the leading causes of cancer‐related morbidity and mortality worldwide. Despite significant advances in surgery, chemoradiotherapy, targeted therapy, and immunotherapy, survival outcomes for patients with advanced gastric cancer remain poor [1, 2]. Metastasis is the major contributor to poor outcomes in gastric cancer, and tumor immune evasion promotes disease progression and limits the efficacy of immunotherapy [3, 4, 5]. Although blockade of the programmed cell death protein 1/programmed cell death ligand 1 (PD‐1/PD‐L1) axis has provided clinical benefit to a subset of patients, the overall response rates remain modest and the durability of responses varies, underscoring the urgent need for combination strategies that simultaneously target tumor‐intrinsic plasticity and reprogram the tumor microenvironment [6, 7].

The coagulation system and tumor biology interact in complex and multifaceted ways. Increasing evidence indicates that coagulation factors exert non‐hemostatic functions that influence tumor cell survival and migration through receptor interactions or downstream kinase signaling cascades [8]. Among these factors, F7 has traditionally been studied in the context of the tissue factor (TF)–FVIIa complex, which activates protease‐activated receptor signaling and promotes tumor progression [9]. Recent studies have shown that components of the coagulation system can regulate immune evasion in pancreatic cancer independently of F7 [10]. In glioblastoma, TF activates tumor‐associated macrophages (TAMs) and promotes extracellular matrix (ECM) remodeling [11]. Similarly, coagulation factor X (FX) independently remodels the immune microenvironment in hepatocellular carcinoma and promotes intrahepatic metastasis [12]. Moreover, autocrine F7 produced by breast cancer cells has been shown to directly promote metastatic progression [13].

However, the expression pattern, functional role, and regulatory mechanisms of F7 in gastric cancer remain largely unclear. In particular, it is unknown whether tumor‐derived F7 functions through autocrine or paracrine mechanisms to promote metastasis and shape an immunosuppressive tumor microenvironment. The coordination between the upstream transcriptional regulation of F7 and downstream signaling pathways also remains to be defined. Our analyses of clinical specimens and multi‐omics datasets revealed that F7 is aberrantly upregulated in metastatic gastric cancer, suggesting that it may contribute to disease progression and represent a potentially druggable therapeutic target.

During the early stages of metastasis, tumor cells often experience anoikis stress [14]. Anoikis resistance is not only a critical step in metastatic adaptation but is also closely linked to immune evasion, ECM remodeling, and abnormal activation of integrin signaling [15, 16]. The ECM provides structural support to maintain cellular homeostasis and plays a key role in cancer metastasis, while its contribution to therapeutic resistance has drawn increasing attention [17]. Notably, transcription factor networks within tumor cells are frequently reprogrammed during metastasis [18]. However, how transcriptional regulation contributes to anoikis resistance, and whether it controls F7 to mediate anoikis resistance and immune‐related effects remain unclear.

Given the limitations of immune checkpoint inhibitor monotherapy, identification of small‐molecule agents that can simultaneously suppress metastasis, alleviate immunosuppression, and synergize with anti–PD‐1 therapy is clinically important. Currently, selective small‐molecule inhibitors targeting F7 are scarce, and their mechanisms of action and potential combination with immune checkpoint blockade remain insufficiently explored.

In this study, we addressed key mechanistic and translational gaps using an integrated approach that combined bioinformatic and clinical data analyses, multiplex immunofluorescence, in vitro anoikis models, tumor–immune co‐culture systems, mouse liver and lymph node metastasis models, immunoprecipitation–mass spectrometry (IP–MS), RNA sequencing (RNA‐seq), chromatin immunoprecipitation (ChIP), and dual‐luciferase reporter assays. We further identified candidate small‐molecule inhibitors through virtual screening, molecular dynamics simulations, and surface plasmon resonance (SPR) and evaluated their efficacy in vitro, in a mouse liver metastasis model, and in patient‐derived xenograft (PDX) models. We found that gastric cancer cells secrete F7, which functions through an autocrine mechanism to activate ITGA2‐associated signaling, thereby promoting metastasis, inducing PD‐L1 expression, and remodeling the extracellular matrix. Under anoikis‐associated stress, hepatocyte nuclear factor 4 alpha (HNF4A) mediates the transcriptional upregulation of F7. Finally, we identified a small‐molecule inhibitor targeting F7 that exhibited antitumor activity and enhanced the efficacy of anti–PD‐1 therapy.

Together, our findings reveal an F7‐centered pathway that links anoikis‐associated transcriptional regulation, ITGA2 signaling, extracellular matrix remodeling, and immune evasion. These results support F7 inhibition, particularly in combination with anti–PD‐1 therapy, as a potential therapeutic strategy for patients with metastatic or immunotherapy‐refractory gastric cancer.

## 2 Materials and Methods

Additional materials and methods are provided in the Supporting Information.

## 3 Results

### 3.1 Is Highly Expressed in Gastric Cancer and Is Associated With Metastasis and Poor Prognosis

To characterize the expression landscape of coagulation factors in gastric cancer, we first analyzed data from The Cancer Genome Atlas (TCGA). Among coagulation factor family members, *F5*, *F7*, and *F12* were markedly upregulated in gastric cancer tissues (Figure S1A). Notably, F7 was identified as a prognostic factor associated with poor overall survival (OS), progression‐free survival (PFS), disease‐free survival (DFS), and disease‐specific survival (DSS) in patients with gastric cancer (Figure S1B). Next, we explored the relationship between coagulation factor–related genes and the immune microenvironment in gastric cancer. Several coagulation factors, including F7, were negatively correlated with immune cell infiltration (Figure S1C,D). These findings are consistent with previous reports showing that the TF–FVIIa complex activates receptor tyrosine kinases involved in cell proliferation, angiogenesis, metastasis, and cancer stemness [19, 20]. Tumor‐derived F7 has also been associated with immune evasion and poor survival in breast cancer [13, 21]. TCGA analysis further showed that F7 expression was markedly elevated in gastric cancer tissues and was significantly associated with poor prognosis (Figure 1A–F). Immunohistochemistry (IHC) analysis in Cohort 2, which included 214 patients with gastric adenocarcinoma, revealed higher F7 expression in tumor tissues than in matched adjacent normal tissues (Figure 1G,H). Further analysis showed that F7 expression was relatively low in non‐metastatic gastric cancer tissues but markedly increased in tumors with lymph node metastasis and in metastatic lymph node lesions, suggesting a potential role for F7 in gastric cancer lymph node metastasis (Figure 1G). Moreover, patients with high F7 expression had significantly shorter overall survival (Figure 1I). In Cohort 1, RT‐qPCR analysis of 32 paired gastric cancer and adjacent non‐tumor tissues showed that *F7* mRNA expression was significantly increased in tumor tissues (Figure 1K), while western blot analysis confirmed higher F7 protein levels (Figure 1J). Clinicopathological analysis of Cohort 2 indicated that F7 expression was associated with T stage, N stage, and lymphovascular invasion (Table 1). Univariate analysis showed poorer outcomes in the high F7 expression group, and multivariate Cox regression analysis identified high F7 expression as an independent prognostic factor for OS in patients with gastric cancer (Figure 1L,M).

**FIGURE 1:**
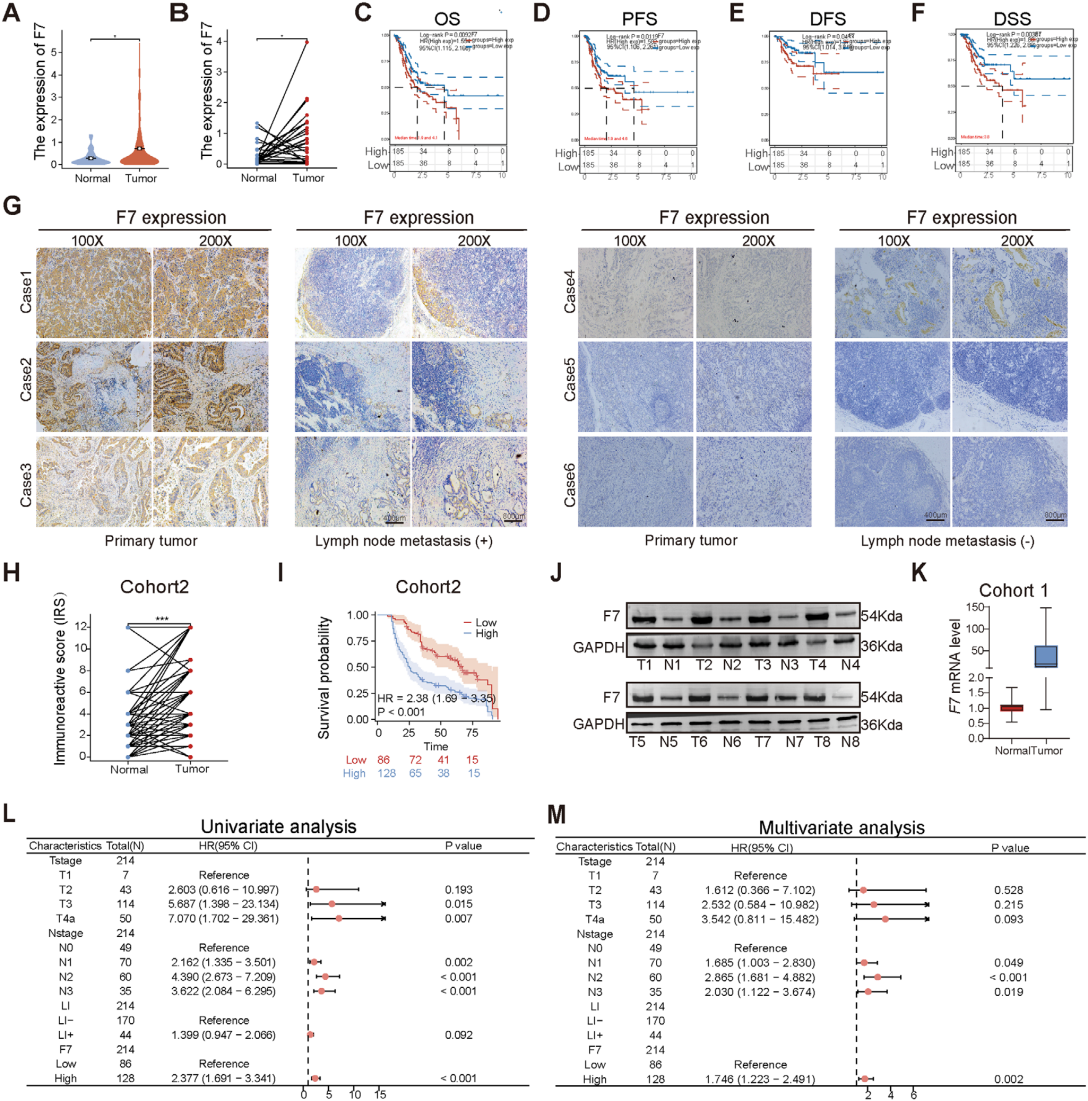
F7 is upregulated in gastric cancer and associated with unfavorable clinical outcomes. (A) Violin plot showing F7 mRNA expression levels in gastric cancer tissues and normal gastric tissues from the TCGA cohort. (B) Paired analysis of matched tumor and adjacent normal tissues demonstrating increased F7 expression in gastric cancer tissues. (C–F) Kaplan–Meier survival analyses comparing overall survival (C), progression‐free survival (D), disease‐free survival (E), and disease‐specific survival (F) between F7‐high and F7‐low expression groups in the TCGA cohort. (G) Representative IHC images showing F7 protein expression in primary gastric cancer tissues from patients with lymph node metastasis, matched metastatic lymph node lesions, and primary tumors from patients without lymph node metastasis. (H) IHC analysis of Cohort 2 showing higher F7 expression in gastric cancer tissues than in matched adjacent normal tissues. (I) Kaplan–Meier survival analysis of Cohort 2 stratified by F7 expression level, showing shorter survival in patients with high F7 expression. (J) Western blot analysis of F7 protein expression in eight paired gastric cancer tissues (T1–T8) and matched adjacent normal tissues (N1–N8). (K) Box plot showing F7 mRNA expression levels in paired gastric cancer and adjacent normal tissues (L,M) Forest plots showing univariate and multivariate Cox regression analyses of overall survival in Cohort 2 (*n* = 214), identifying F7 expression as an independent prognostic factor. *p < 0.05; **p < 0.01; ***p < 0.001; ****p < 0.0001.

**TABLE 1:** Baseline Characteristics of Patients with Gastric Cancer.

Characteristics	Low	High	*p* value
n	86	128	
sex, n (%)			0.481
1	51 (23.8%)	82 (38.3%)	
2	35 (16.4%)	46 (21.5%)	
age, n (%)			0.607
2	59 (27.6%)	92 (43%)	
1	27 (12.6%)	36 (16.8%)	
Tstage, n (%)			0.005
T1	6 (2.8%)	1 (0.5%)	
T2	23 (10.7%)	20 (9.3%)	
T3	36 (16.8%)	78 (36.4%)	
T4a	21 (9.8%)	29 (13.6%)	
Nstage, n (%)			< 0.001
N0	34 (15.9%)	15 (7%)	
N1	32 (15%)	38 (17.8%)	
N2	12 (5.6%)	48 (22.4%)	
N3	8 (3.7%)	27 (12.6%)	
stage, n (%)			< 0.001
1A	6 (2.8%)	1 (0.5%)	
1B	14 (6.5%)	2 (0.9%)	
2A	15 (7%)	16 (7.5%)	
2B	24 (11.2%)	38 (17.8%)	
3A	18 (8.4%)	39 (18.2%)	
3B	8 (3.7%)	26 (12.1%)	
3C	1 (0.5%)	6 (2.8%)	
vascular invasion, n (%)			0.021
LI‐	75 (35%)	95 (44.4%)	
LI+	11 (5.1%)	33 (15.4%)	
state, n (%)			< 0.001
live	37 (17.3%)	17 (7.9%)	
Dead	49 (22.9%)	111 (51.9%)	

### 3.2 Promotes Anoikis Resistance, Invasion, and Metastasis in Gastric Cancer Cells

To evaluate the role of F7 in anoikis resistance, we established a suspension‐culture model using gastric cancer cells. Stable F7‐knockdown (sh‐F7) and F7‐overexpression (oe‐F7) cell lines were generated by lentiviral transduction (Figure S2A). F7 protein levels in cell culture supernatants were measured by ELISA. F7 secretion was markedly decreased in F7 knockdown cells, whereas F7 overexpression led to a corresponding increase in secreted F7 levels, indicating that changes in intracellular F7 expression were reflected in its secretion (Figure S2B). Functional assays further showed that F7 knockdown in MKN45 gastric cancer cells significantly reduced anoikis resistance, invasion, and migration (Figure 2A–C). Conversely, F7 overexpression in MKN45 cells enhanced anoikis resistance, invasion, and migration (Figure S2C–E). Similarly, F7 overexpression in HGC27 cells promoted anoikis resistance, invasion, and migration (Figure 2A–C). Given that F7 is a secreted protein, we next investigated whether it functions in an autocrine manner. In F7‐silenced cells, treatment with recombinant F7 (rF7) restored anoikis resistance as well as invasive and migratory capacities (Figure S2H–J).

**FIGURE 2:**
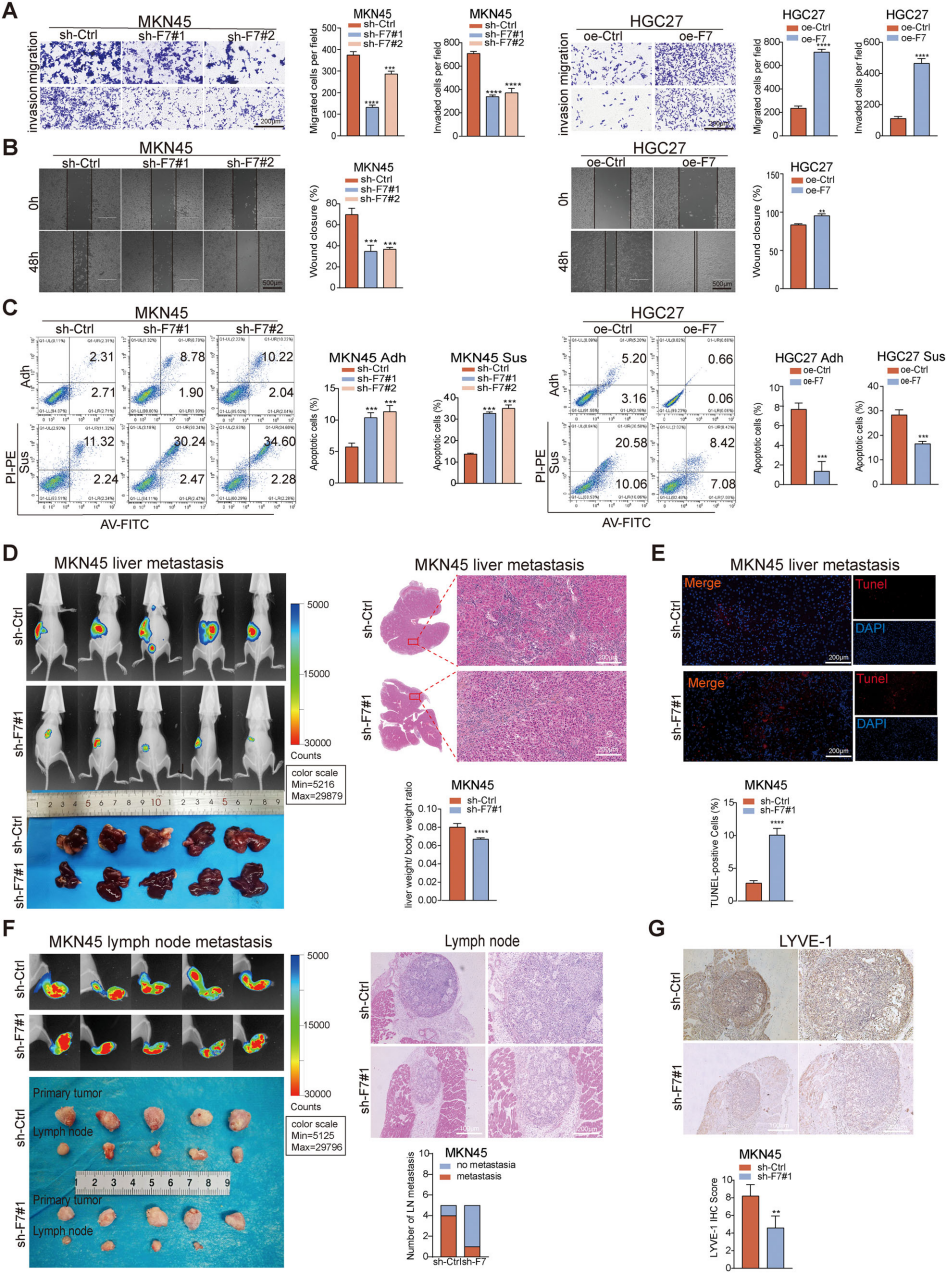
F7 promotes anoikis resistance, migration, invasion, and metastasis of gastric cancer cells (A‐B) Transwell migration/invasion assays and wound‐healing assays showing the effects of F7 expression alteration on the migratory and invasive abilities of gastric cancer cells. (C) Flow cytometric analysis of apoptosis in gastric cancer cells under adherent and suspension conditions. F7 knockdown (sh‐F7#1/#2) in MKN45 cells increased apoptosis, whereas F7 overexpression (oe‐F7) in HGC27 cells decreased apoptosis. (D) In vivo liver metastasis model established by intrasplenic injection of MKN45 cells. Representative bioluminescence imaging (BLI), gross liver images, and hematoxylin and eosin (H&E) staining show reduced metastatic lesions following F7 knockdown. Quantification of liver metastatic burden based on the liver weight/body weight ratio is shown. (E) Representative TUNEL staining of liver metastatic lesions showing increased apoptotic cells in the sh‐F7#1 group. (F) In vivo lymph node metastasis model established by footpad injection of MKN45 cells. Representative BLI and H&E staining demonstrate reduced lymph node metastasis following F7 knockdown. The incidence of lymph node metastasis is summarized by stacked bar graphs. (G) Representative immunohistochemical staining for LYVE‐1 in lymph node metastatic lesions showing reduced LYVE‐1‐positive lymphatic vessels following F7 knockdown. *p < 0.05; **p < 0.01; ***p < 0.001; ****p < 0.0001.

To investigate the metastatic potential of F7 in vivo, we used established liver and footpad lymph node metastasis models [12, 22]. Stable F7‐knockdown or control shRNA (sh‐Ctrl) MKN45 cells were injected into the spleens of NOD–SCID mice (*n* = 5 per group), and liver metastasis was evaluated after 4 weeks. For the lymph node metastasis model, MKN45 cells were injected into the footpads of NOD–SCID mice (*n* = 5 per group), and tumors in the footpads and popliteal lymph nodes were examined after 4 weeks. F7 knockdown significantly reduced the incidence of liver and lymph node metastases (Figure 2D,F). TUNEL staining of liver metastatic lesions showed increased apoptosis in the sh‐F7 group (Figure 2E). LYVE‐1 staining in lymph node metastatic lesions was reduced in the sh‐F7 group (Figure 2G). In the liver metastasis model, F7 overexpression in MKN45 cells enhanced liver metastatic capacity (Figure S2F). TUNEL staining further showed that the proportion of apoptotic cells in liver metastatic lesions was reduced after F7 overexpression (Figure S2G). Taken together, these findings indicate that F7 promotes anoikis resistance and metastatic phenotypes in gastric cancer.

### 3.3 Loss of Tumor‐Derived F7 Is Associated With Enhanced CD8^+^ T‐Cell Infiltration and Cytotoxic Function

To assess the role of F7 in shaping the immune microenvironment of gastric cancer, we first examined its association with immune cell infiltration in the TCGA dataset. Using the Tumor Immunophenotype (TIP) framework, we found that F7 expression was inversely associated with T‐cell infiltration and cytolytic activity in the cancer‐immunity cycle (Figure S3A). xCell analysis revealed that F7 expression was negatively correlated with multiple immune cell populations, with the strongest inverse association observed for CD8^+^ T cells (Figure S3B). Consistently, tumors with low F7 expression exhibited higher CD8^+^ T‐cell infiltration than tumors with high F7 expression (Figure S3C). To validate these findings, we analyzed 64 gastric cancer specimens using IHC and multiplex immunofluorescence (mIF). IHC analysis showed significantly fewer CD8^+^ T cells in the high‐F7 group, CD4^+^ T‐cell infiltration showed a decreasing trend without reaching statistical significance, whereas tumor‐associated macrophage (TAM) abundance did not differ between groups (Figure S3D). mIF further showed that the high‐F7 group had fewer CD8^+^ T cells and reduced GZMB expression, indicating impaired cytotoxic activity of infiltrating CD8^+^ T cells (Figure S3E).

To further evaluate the functional association between F7 and CD8^+^ T‐cell activity, human peripheral blood mononuclear cells (PBMCs) were isolated, activated, and co‐cultured with gastric cancer cells carrying F7 knockdown (sh‐F7), F7 overexpression (oe‐F7), or their respective controls (sh‐Ctrl and oe‐Ctrl). F7 knockdown increased the proportion of CD8^+^ T cells, whereas F7 overexpression significantly reduced it (Figure 3B). In contrast, CD4^+^ T cells proportions remained largely unaffected (Figure S4A). The levels of GZMB, perforin, and IFN‐γ mirrored the changes in CD8^+^ T cells, increasing after F7 knockdown and decreasing after F7 overexpression (Figure 3C,D). Because F7 is a secreted protein, we next examined whether it directly modulates T‐cell activity in a tumor cell–dependent manner. PBMCs were cultured with or without HGC27 cells and treated with rF7 at 0, 20, 40, and 80 ng mL^−1^. Under co‐culture conditions, the proportion of CD8^+^ T cells decreased in a dose‐dependent manner after rF7 treatment, whereas no significant change was observed in the absence of HGC27 cells (Figure S4B,C). Taken together, these results indicate that F7 suppresses CD8^+^ T‐cell abundance and effector function through a tumor cell–dependent mechanism.

**FIGURE 3:**
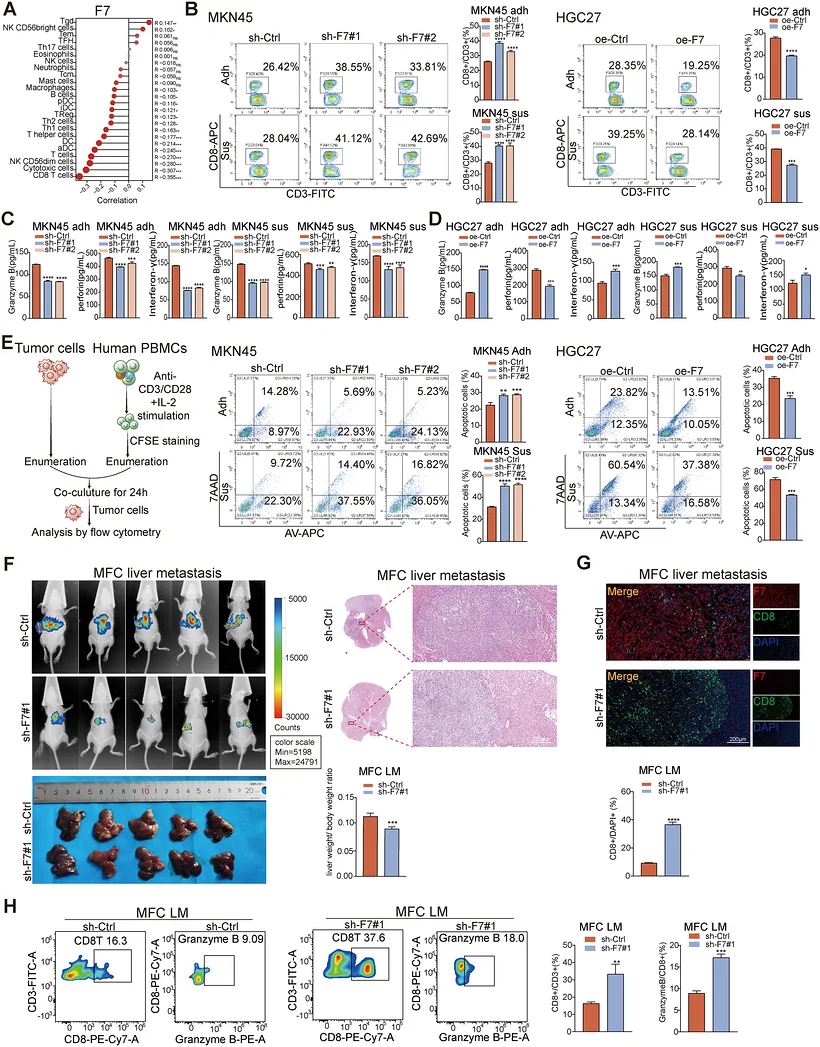
Loss of tumor‐derived F7 is associated with enhanced CD8^+^ T‐cell infiltration and cytotoxic activity. F7 weakens CD8^+^ T‐cell activity and promotes metastasis. (A) Spearman correlation analysis between F7 expression and immune cell signatures in the TCGA cohort. (B) Flow cytometric analysis of CD8^+^ T cells in PBMC–gastric cancer cell co‐culture systems under adherent (Adh) and suspension (Sus) conditions. MKN45 cells with F7 knockdown (sh‐F7#1/#2) and HGC27 cells with F7 overexpression (oe‐F7) were compared with their corresponding controls. Quantification of CD8^+^ T‐cell percentages is shown. (C,D) ELISA analysis of granzyme B, perforin, and IFN‐γ levels in culture supernatants from MKN45 (C) and HGC27 (D) co‐culture systems. F7 knockdown increased, whereas F7 overexpression decreased, cytotoxic effector molecule release. (E) Schematic illustration of the tumor cell–PBMC co‐culture assay. Flow cytometric analysis showing increased tumor cell apoptosis after co‐culture with PBMCs following F7 knockdown, whereas F7 overexpression reduced T‐cell–mediated tumor cell apoptosis. (F) Immunocompetent mouse liver metastasis model established using MFC cells. Representative bioluminescence imaging, gross liver images, and H&E staining showing reduced metastatic lesions in the sh‐F7 group. (G) Representative immunofluorescence staining of CD8 and F7 in liver metastatic lesions, showing increased CD8^+^ T‐cell infiltration following F7 knockdown. (H) Flow cytometric analysis of metastatic liver tissues from the MFC model showing increased proportions of CD8^+^ T cells and granzyme B‐positive CD8^+^ T cells in the sh‐F7 group. *p < 0.05; **p < 0.01; ***p < 0.001; ****p < 0.0001.

Next, we examined whether tumor‐derived F7 affects T‐cell‐mediated cytotoxicity. After T‐cell–tumor cell co‐culture, F7 knockdown increased T‐cell–mediated apoptosis of gastric cancer cells under both adherent (Adh) and suspension (Sus) conditions, whereas F7 overexpression decreased T‐cell–mediated killing (Figure 3E). In an immunocompetent syngeneic liver metastasis model using MFC cells, F7 knockdown reduced metastatic tumor burden (Figure 3F). Immunofluorescence analysis showed increased CD8^+^ T‐cell infiltration within metastatic lesions (Figure 3G). Flow cytometric analysis further confirmed an increased proportion of CD8^+^ T cells and a higher fraction of GZMB‐positive CD8^+^ T cells (Figure 3H), whereas no significant change in regulatory T cells was observed (Figure S4D). Collectively, these findings suggest that F7 is associated with reduced CD8^+^ T‐cell infiltration and impaired cytotoxic activity. To further determine whether F7 promotes gastric cancer metastasis by inhibiting CD8^+^ T‐cell‐mediated antitumor immunity, we depleted CD8^+^ T cells in the F7 knockdown mouse liver metastasis model. CD8^+^ T‐cell depletion significantly increased the number of liver metastatic lesions compared with the F7‐knockdown group, although the metastatic burden remained lower than that in the control group (Figure S4F). Flow cytometric analysis of peripheral blood confirmed successful depletion of CD8^+^ T cells in the depletion group, and immunofluorescence staining further showed little to no CD8^+^ T‐cell infiltration in tumor tissues after CD8^+^ T‐cell depletion (Figure S4G,H). Together, these data show that F7 suppresses the abundance and effector function of CD8^+^ T cells both in vitro and in vivo, thereby contributing to an immunosuppressive tumor microenvironment. Moreover, F7 may promote gastric cancer metastasis, at least partly, by suppressing the infiltration and function of CD8^+^ T cells.

### 3.4 Autocrine F7 Interacts With ITGA2 to Promote Gastric Cancer Metastasis

To elucidate the downstream mechanisms by which F7 promotes gastric cancer metastasis, we performed RNA‐seq and IP–MS analyses. Transcriptomic pathway enrichment analysis identified ECM–receptor interaction, PI3K–AKT signaling, transcriptional dysregulation in cancer, and cell adhesion molecules as the most significantly enriched pathways (Figure S5A–C). IP–MS analysis identified ITGA2 as a potential F7‐interacting protein (Figure S5D), and subsequent pathway enrichment analysis further highlighted ECM–receptor interaction–related pathways (Figure S5E,F). LC–MS/MS analysis of the F7 protein complex detected ITGA2 specific peptides in the fragmentation spectra (Figure 4A), supporting ITGA2 as a candidate F7‐binding partner. To validate this interaction, we performed reciprocal Co‐IP assays in cells overexpressing HA‐tagged ITGA2 (HA‐ITGA2) and FLAG‐tagged F7 (FLAG‐F7). Anti‐HA pulldown recovered FLAG‐F7 and, conversely, anti‐FLAG pulldown recovered HA‐ITGA2. Whole‐cell lysates confirmed the expression of both proteins, with β‐actin used as the loading control (Figure 4B). At the endogenous level, immunoprecipitation using anti‐F7 or anti‐ITGA2 antibodies in MKN45 cells pulled down the reciprocal protein, whereas IgG controls showed no detectable interaction, supporting the interaction under physiological expression conditions (Figure 4C). IF colocalization analysis showed clear overlap between F7 and ITGA2 at the plasma membrane and pericellular regions of MKN45 cells (Figure 4F). IF analysis of gastric cancer tissue specimens also showed colocalization of F7 and ITGA2 (Figure 4D). Taken together, these results indicate that F7 interacts with ITGA2 in gastric cancer cells.

**FIGURE 4:**
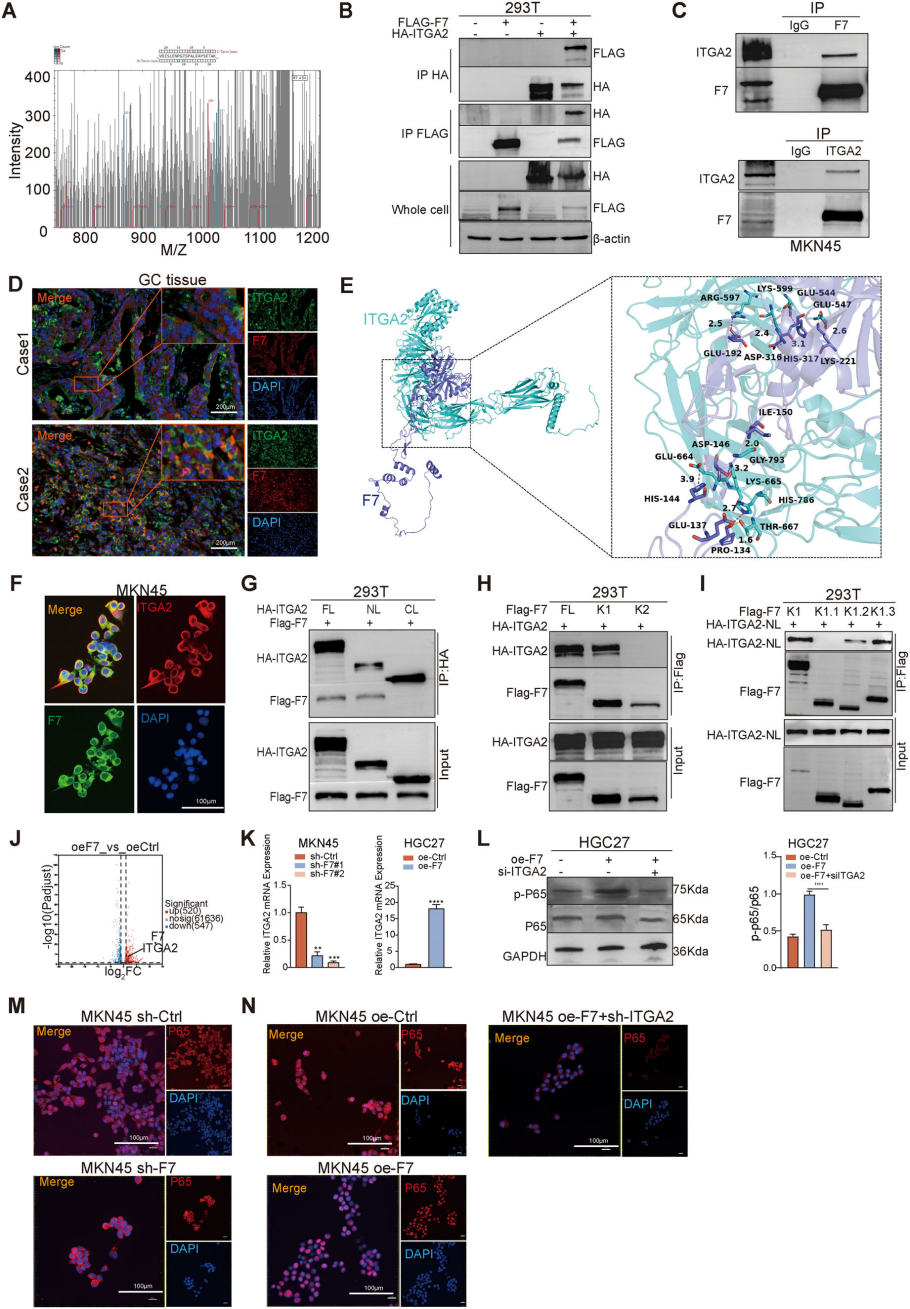
F7 interacts with ITGA2 to activate downstream FAK–NF‐κB signaling and promote gastric cancer metastasis. (A) LC–MS analysis identifying ITGA2 as a potential F7‐interacting protein. (B) Reciprocal Co‐IP assays confirming the interaction between F7 and ITGA2 in 293T cells. (C) Endogenous Co‐IP confirming the interaction between F7 and ITGA2 in MKN45 cells. (D) Representative immunofluorescence images from two gastric cancer patient samples showing co‐expression of F7 and ITGA2. F7 is shown in red, ITGA2 in green, and nuclei are counterstained with DAPI (blue). The boxed regions are shown at higher magnification in the corresponding inset images. (E) Predicted docking model of the F7–ITGA2 interface with key contact residues. (F) Immunofluorescence analysis showing colocalization of F7 and ITGA2 in MKN45 cells. F7 is shown in green, ITGA2 in red, and nuclei are counterstained with DAPI (blue). (G) Co‐IP of ITGA2 full‐length (FL), N‐terminal large (NL), and C‐large (CL) with F7, indicating that F7 primarily binds to the NL region of ITGA2. (H) Co‐IP of full‐length and truncated FLAG‐F7 fragments with HA‐ITGA2 in cotransfected 293T cells. (I) Fine mapping with F7‐K1 subfragments (K1.1, K1.2, K1.3), and HA‐ITGA2‐NL identifying the critical binding region. (J) Volcano plot of differential expression highlights ITGA2 as significantly upregulated following F7 overexpression. (K) RT‐qPCR validation showing that F7 modulates ITGA2 mRNA expression in MKN45 and HGC27 cells. (L) Western blot analysis showing that p65 phosphorylation is regulated by the F7–ITGA2 axis in HGC27 cells. (M,N) Immunofluorescence analyses demonstrating that p65 nuclear translocation depends on F7–ITGA2 signaling in gastric cancer cells. p65 is shown in red, and nuclei are counterstained with DAPI (blue). *p < 0.05; **p < 0.01; ***p < 0.001; ****p < 0.0001.

Molecular docking analysis predicted multiple hydrogen‐bonding and electrostatic interactions between F7 and ITGA2, with a docking score of −744, supporting the presence of potential binding interfaces between these two proteins (Figure 4E). The amino acid residues involved in the predicted F7–ITGA2 interaction are summarized in Table S3. In the docking model, F7 is displayed as a dark blue cartoon structure, whereas ITGA2 is displayed as a cyan cartoon structure. Notably, the predicted major binding interface of F7 was mainly located within the K1.2 and K1.3 regions, while both the NL and CL regions of ITGA2 appeared to provide potential contact sites for F7 binding. To further map the interaction regions, we generated truncation constructs for ITGA2 and F7, including ITGA2‐FL (aa 1–1181), ITGA2‐NL (aa 1–630), and ITGA2‐CL (aa 631–1181); F7‐FL (aa 61–466), F7‐K1 (aa 61–212), F7‐K2 (aa 212–466), and the K1 sub‐fragments K1.1 (aa 61–105), K1.2 (aa 106–145), and K1.3 (aa 146–212) (Figure S5M). Co‐IP using anti‐HA antibody showed that F7 primarily associated with ITGA2‐NL, whereas no detectable interaction was observed between F7 and ITGA2‐CL (Figure 4G). Co‐IP assays using anti‐FLAG antibody showed that F7‐FL interacted with ITGA2. F7‐K1 retained binding to ITGA2, whereas F7‐K2 showed markedly reduced or undetectable binding (Figure 4H). Further Co‐IP assays of the K1 sub‐fragments revealed that K1.2 and K1.3 retained binding activity with ITGA2‐NL (Figure 4I). Although molecular docking predicted potential contact sites in both ITGA2‐NL and ITGA2‐CL, the Co‐IP results indicate that the experimentally detectable F7–ITGA2 interaction is primarily mediated by ITGA2‐NL. We next examined whether F7 regulates ITGA2‐associated downstream signaling. In MKN45 cells, F7 knockdown reduced ITGA2 protein expression and decreased FAK and AKT phosphorylation. Conversely, in HGC27 cells, F7 overexpression increased ITGA2 expression and enhanced FAK and AKT phosphorylation, indicating that F7 activates ITGA2‐associated FAK–AKT signaling (Figure S5H).

To determine whether the effects of F7 on anoikis resistance, migration, and invasion depend on ITGA2, we overexpressed F7 in HGC27 cells while simultaneously silencing ITGA2 with siRNA. Western blot analysis showed that F7 overexpression upregulated ITGA2 expression and markedly increased phosphorylation levels of FAK and AKT. Moreover, ITGA2 silencing in F7‐overexpressing cells abolished the increases in p‐FAK and p‐AKT levels (Figure S5I). Apoptosis assays further showed that ITGA2 silencing partially reversed the anti‐apoptotic effect of F7 overexpression (Figure S5L). Migration and invasion assays similarly showed that ITGA2 silencing substantially attenuated the pro‐migratory and pro‐invasive effects of F7 overexpression (Figure S5J,K). IF analysis of liver metastatic lesions formed by MKN45 cells demonstrated that sh‐Ctrl tumors exhibited colocalized F7 and ITGA2 signals within tumor cells, whereas sh‐F7 tumors showed markedly reduced F7 expression and was accompanied by decreased ITGA2 staining (Figure S5G). Taken together, these results indicate that F7 promotes gastric cancer cell anoikis resistance, migration, and invasion at least partly through ITGA2‐mediated activation of FAK–AKT signaling.

To examine how F7 regulates ITGA2 expression, RNA‐seq analysis of F7‐overexpressing cells showed increased ITGA2 expression (Figure 4J) and enrichment of NF‐κB signaling (Figure S5C). Previous studies have shown that phosphorylated FAK facilitates NF‐κB nuclear translocation, supporting a mechanistic link between FAK activation and NF‐κB‐mediated transcriptional regulation [23]. In addition, NF‐κB has been reported to transcriptionally activate ITGA2 [24, 25]. Consistent with these findings, *ITGA2* mRNA expression decreased after F7 knockdown and increased after F7 overexpression (Figure 4K). Moreover, F7 overexpression promoted NF‐κB activation, while ITGA2 silencing attenuated this effect in F7‐overexpressing cells (Figure 4L). Consistently, F7 knockdown reduced, whereas F7 overexpression enhanced, NF‐κB nuclear translocation; this increase was suppressed by ITGA2 silencing (Figure 4M,N). These findings suggest that F7 activates NF‐κB signaling, at least partly through ITGA2.

Collectively, these results indicate that autocrine F7 interacts with ITGA2 and promotes FAK phosphorylation, and activates downstream NF‐κB signaling. NF‐κB may then transcriptionally upregulate ITGA2, thereby forming an F7–ITGA2–FAK–NF‐κB positive feedback loop. This signaling axis amplifies ITGA2‐associated signaling and promotes anoikis resistance, migration, invasion, and metastasis in gastric cancer.

### 3.5 Regulates PD‐L1 Expression and Collagen Remodeling Through ITGA2 to Impair CD8^+^ T‐Cell Immunity

To characterize the distribution of F7‐associated cell populations and their intercellular communication within the tumor microenvironment, we calculated an F7 signature score using single‐cell transcriptomic data and performed ligand–receptor interaction analyses. Among all cell types, the F7 expression score was most enriched in epithelial cells (Figure S6A). Further subclustering of the epithelial compartment showed higher F7 expression in tumor‐derived epithelial cells than in normal epithelial cells (Figure S6B). Cell–cell communication analysis indicated that F7^+^ epithelial cells exhibited frequent and strong interactions with T cells and cancer‐associated fibroblasts, suggesting F7^+^ epithelial cells may be involved in immune regulation and ECM remodeling (Figure S6C–F).

Given that immune checkpoint signaling represents a key mechanism for tumor immune evasion [26], we next investigated whether F7 regulates PD‐L1 expression. RNA‐seq revealed that PD‐L1 (*CD274*) mRNA levels increased following F7 overexpression (Figure S5A). Given that ITGA2 has been implicated in PD‐L1 regulation [27], we performed enrichment analysis of seven canonical signaling pathways associated with PD‐L1 regulation in tumor cells [28]. Among these, PI3K–AKT signaling showed the strongest enrichment in high‐F7‐expressing tumor cells (Figure 5A). Flow cytometric and western blot analyses confirmed that PD‐L1 expression decreased in F7 knockdown and increased upon F7 overexpression (Figure 5B,C). In HGC27 cells, F7 overexpression increased ITGA2 and PD‐L1 expression and enhanced PI3K and AKT phosphorylation. Moreover, ITGA2 silencing in F7‐overexpressing cells partially reversed the increases in p‐PI3K, p‐AKT, and PD‐L1 levels, suggesting that F7‐induced PD‐L1 upregulation is partly dependent on ITGA2‐mediated PI3K–AKT signaling (Figure 5D). Moreover, treatment with the PI3K–AKT pathway inhibitor (LY294002) partially reduced PD‐L1 expression in F7‐overexpressing cells, further supporting the involvement of PI3K–AKT signaling in F7‐mediated PD‐L1 regulation (Figure 5E). In co‐culture experiments with human T cells, F7 overexpression reduced the proportion of CD3^+^CD8^+^ T cells under both Adh and Sus conditions. PD‐L1 blockade partially rescued the reduction in CD8^+^ T‐cell proportion induced by F7 overexpression, whereas isotype IgG control exerted no statistically significant effect (Figure 5F). Consistently, ITGA2 silencing in F7‐overexpressing cells partially restored the proportion of CD8^+^ T cells (Figure 5G). Following co‐culture, tumor cell apoptosis was reduced by F7 overexpression under both adherent and suspension conditions, whereas ITGA2 silencing partially reversed this anti‐apoptotic effect (Figure 5H). Taken together, these findings indicate that F7‐mediated ITGA2 signaling contributes to PD‐L1 upregulation and impairs CD8^+^ T‐cell abundance and cytotoxic function.

**FIGURE 5:**
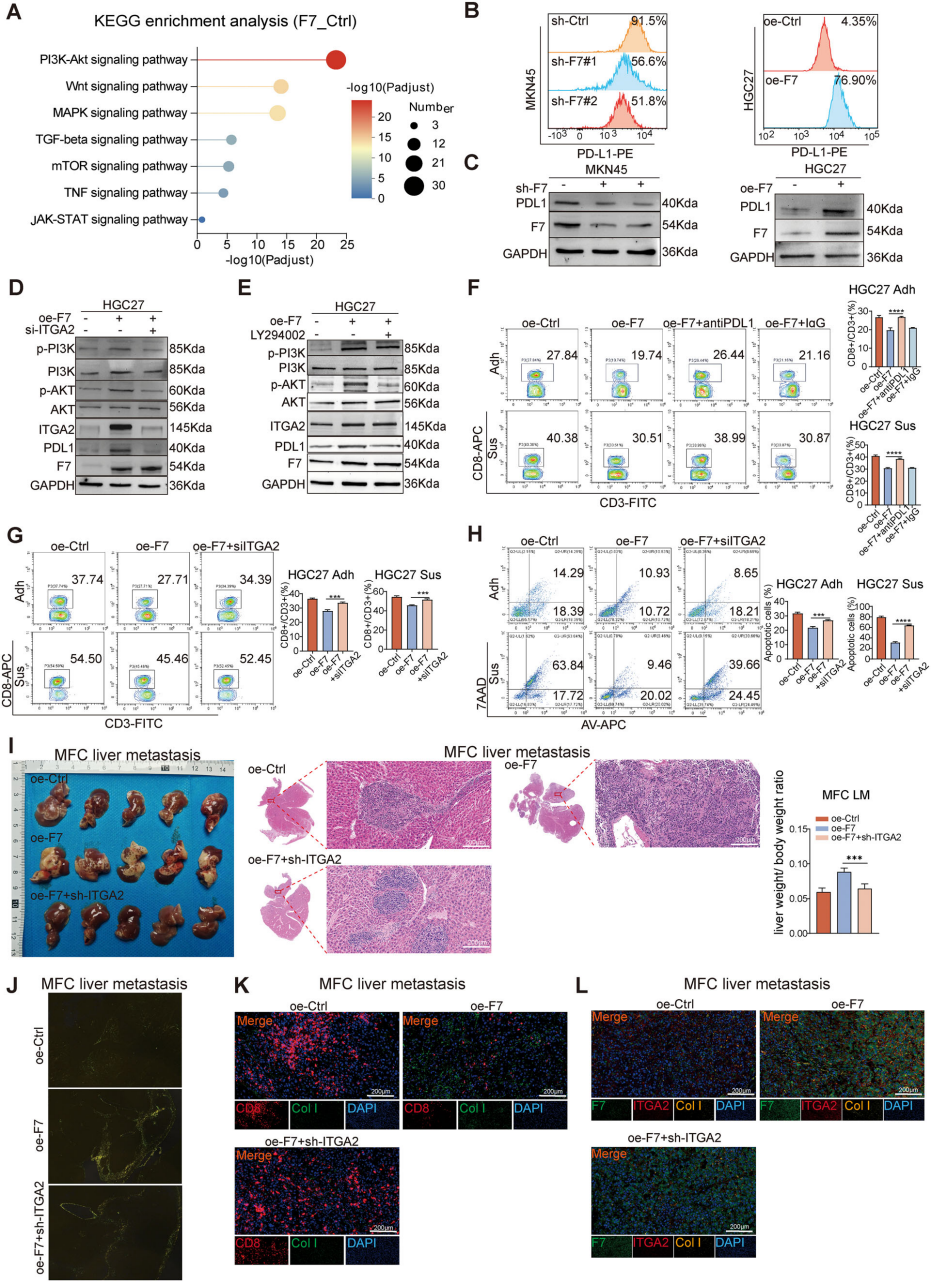
F7 regulates PD‐L1 expression and collagen remodeling through ITGA2‐associated signaling. (A) KEGG pathway enrichment of genes altered by F7 overexpression compared with control cells. The enriched pathways include PI3K–AKT, Wnt, MAPK, TGF‐β, mTOR, TNF, and JAK–STAT signaling pathways. (B,C) Flow cytometry and western blot analyses showing increased PD‐L1 expression in HGC27 with F7 overexpression and decreased PD‐L1 in MKN45 cells following F7 knockdown. (D) Western blot analyses showing that F7 overexpression increased ITGA2 expression and enhanced PI3K and AKT phosphorylation, whereas ITGA2 silencing attenuated these effects. (E) Western blot analysis showing that treatment with the PI3K–AKT pathway inhibitor LY294002 partially reduced PD‐L1 expression in F7‐overexpressing cells. (F) Flow cytometric analysis showing that F7 overexpression reduced the proportion of CD8^+^ T cells, whereas PD‐L1 blockade partially restored CD8^+^ T‐cell abundance. (G,H) Flow cytometry quantification of CD8^+^ T cells and tumor cell apoptosis in co‐culture experiments demonstrating that ITGA2 knockdown partially rescued CD8^+^ T‐cell abundance and T‐cell–mediated tumor cell apoptosis in F7‐overexpressing cells. (I) MFC liver metastasis model showing that F7 overexpression increased metastatic burden, whereas ITGA2 knockdown reduced F7‐induced liver metastasis. (J) Sirius Red staining of liver metastatic lesions showing increased collagen deposition following F7 overexpression and partial reduction after ITGA2 knockdown. (K,L) Immunofluorescence staining of liver metastatic lesions showing that F7 overexpression increased collagen I deposition and was associated with reduced CD8^+^ T‐cell infiltration, whereas ITGA2 knockdown reduced collagen I deposition and partially restored CD8^+^ T‐cell infiltration. *p < 0.05; **p < 0.01; ***p < 0.001; ****p < 0.0001.

Previous sequencing results indicated that F7 knockdown affects ECM‐related pathways, and ECM remodeling has been implicated in regulating CD8^+^ T‐cell infiltration. Our earlier findings further showed that F7 promotes gastric cancer metastasis and immune evasion through ITGA2‐associated signaling pathways. To further investigate the role of ITGA2 in F7‐mediated metastasis, ITGA2 silencing in F7‐overexpressing cells reduced the number of liver metastatic lesions compared with F7 overexpression alone (Figure 5I). As a member of the integrin family, ITGA2 has been implicated in ECM remodeling. Sirius Red staining showed that F7 overexpression increased collagen deposition, whereas ITGA2 silencing partially reduced collagen accumulation (Figure 5J). Given that collagen accumulation has been associated with altered T‐cell infiltration [29], we further examined the spatial relationship between CD8^+^ T cells and collagen I by IF. F7 overexpression markedly increased collagen I deposition which was associated with reduced CD8^+^ T‐cell infiltration. In contrast, ITGA2 knockdown decreased collagen I deposition and partially restored CD8^+^ T‐cell infiltration (Figure 5K). F7 overexpression increased collagen I deposition, whereas ITGA2 knockdown reduced collagen I deposition, indicating that F7 promotes Collagen I remodeling through ITGA2‐associated signaling (Figure 5L). These results suggest that F7 promotes ITGA2‐associated collagen remodeling, which is associated with reduced CD8^+^ T‐cell infiltration and may contribute to immune evasion in gastric cancer.

### 3.6 HNF4A Transcriptionally Activates F7 Expression in Gastric Cancer and Under Anoikis Stress

Given the elevated F7 mRNA levels observed in gastric tumors, we hypothesized that F7 expression may be controlled by upstream transcriptional regulators in gastric cancer. By integrating multiple databases to screen potential transcription factors of F7 (Figure 6A), we identified five putative upstream regulators. Among these candidates, bioinformatic analyses showed that HNF4A was highly expressed in gastric cancer and that elevated HNF4A expression predicted poor prognosis (Figure S7). Across public transcriptomic datasets, HNF4A expression showed a strong positive correlation with F7 expression (Figure 6B). IHC of gastric cancer tissues further confirmed high HNF4A and F7 expression within the tumor epithelium, with their protein levels significantly correlated within the same specimens (Figure 6C).

**FIGURE 6:**
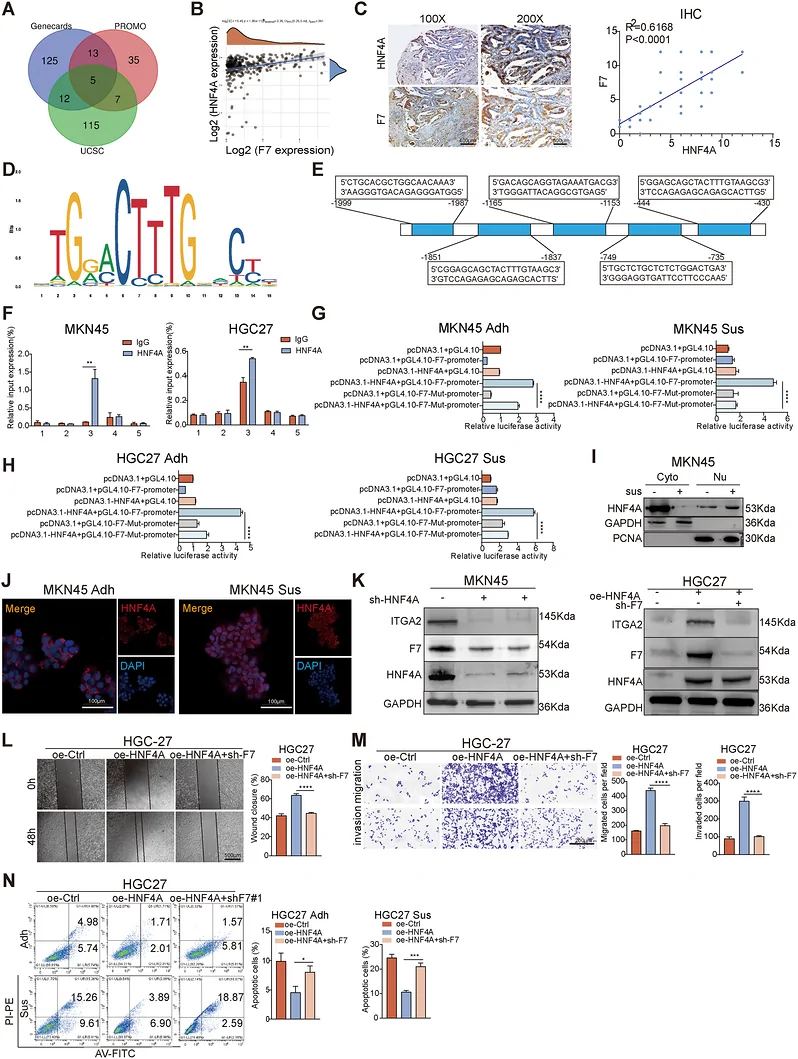
HNF4A transcriptionally activates F7 expression and promotes malignant phenotypes in gastric cancer. (A) Venn diagram showing overlapping candidate transcription factors of F7 predicted by multiple databases. (B) Correlation analysis showing a positive association between HNF4A and F7 expression. (C) Representative IHC images showing the co‐expression of HNF4A and F7 in gastric cancer tissues. (D) Sequence logo of the predicted HNF4A‐binding motif within the F7 promoter region. (E) Schematic illustration of the F7 promoter region showing predicted HNF4A‐binding sites. (F) ChIP–qPCR analysis showing HNF4A enrichment at the F7 promoter in MKN45 and HGC27 cells. (G,H) Dual‐luciferase reporter assays using wild‐type and mutant F7 promoters showing that HNF4A activates F7 transcription under adherent (Adh) and suspension (Sus) conditions. (I) Western blot analysis showing increased nuclear HNF4A and decreased cytoplasmic HNF4A levels under suspension culture conditions. (J) Immunofluorescence analysis of MKN45 cells showing HNF4A localization under Adh and Sus conditions. (K) Western blot analysis showing that HNF4A knockdown or overexpression modulates F7 and ITGA2 protein expression. (L–N) Wound ‐healing, apoptosis, and Transwell migration/invasion assays in HGC27 cells showing that HNF4A overexpression promotes migration, invasion, and anoikis resistance, whereas F7 knockdown attenuates these effects. *p < 0.05; **p < 0.01; ***p < 0.001; ****p < 0.0001.

JASPAR motif analysis identified potential HNF4A‐binding motifs consistent with the canonical HNF4A recognition sequence (Figure 6D), and multiple candidate HNF4A‐binding sites were predicted within the F7 promoter region (Figure 6E). To determine whether HNF4A directly binds to and regulates F7 transcription, we performed ChIP–qPCR and dual‐luciferase reporter assays. ChIP–qPCR demonstrated significant HNF4A enrichment at predicted site 3 within the F7 promoter in MKN45 and HGC27 cells compared with IgG controls (Figure 6F), supporting the binding of HNF4A to the F7 promoter region. Using a dual‐luciferase reporter assay, HNF4A overexpression significantly increased F7 promoter activity under both adherent and suspension conditions, whereas mutation of the HNF4A‐binding motif markedly reduced this transcriptional activation in both cell lines (Figure 6G,H). Finally, IF analysis confirmed that HNF4A was predominantly localized in the nucleus and exhibited enhanced nuclear accumulation under suspension conditions, consistent with the western blot results (Figure 6I,J).

Collectively, these findings indicate that HNF4A binds to the F7 promoter and promotes F7 transcription, while anoikis stress enhances F7 expression through increased HNF4A nuclear accumulation. To further define the functional role of HNF4A in gastric cancer, we generated stable HNF4A knockdown (sh‐HNF4A) and overexpression (oe‐HNF4A) cell lines and performed in vitro and in vivo functional assays. Compared with control cells, HNF4A overexpression enhanced anoikis resistance, whereas HNF4A knockdown produced the opposite effect (Figure S8A). Similarly, Transwell and wound‐healing assays showed that HNF4A overexpression enhanced, whereas HNF4A knockdown reduced the invasive and migratory capacities of MKN45 and HGC27 cells (Figure S8B,C). In mouse models of liver and footpad lymph node metastases established as described for the F7 experiments, HNF4A knockdown markedly reduced liver and lymph node metastatic burden (Figure S8D,F). Correspondingly, TUNEL staining of liver lesions revealed increased apoptosis in the sh‐HNF4A group (Figure S8E), and LYVE‐1 staining in lymph node metastases was decreased (Figure S8G). These findings indicate that HNF4A promotes metastatic phenotypes in gastric cancer.

To verify the functional impact of the HNF4A–F7 axis on gastric cancer malignancy, we assessed protein expression and performed functional assays in MKN45 and HGC27 cells. In MKN45 cells, HNF4A knockdown reduced F7 and ITGA2 protein levels, whereas HNF4A overexpression increased their expression in HGC27 cells. Moreover, F7 knockdown in HNF4A‐overexpressing cells reduced ITGA2 upregulation, indicating that HNF4A regulates ITGA2 expression, at least partly, through F7 (Figure 6K). To determine whether the oncogenic effects of HNF4A depend on F7, HGC27 cells with HNF4A overexpression combined with F7 knockdown were analyzed. Apoptosis assays showed that F7 knockdown partially reversed the anti‐apoptotic effect induced by HNF4A overexpression (Figure 6N). Migration and invasion assays demonstrated that F7 knockdown significantly attenuated the pro‐migratory and pro‐invasive effects of HNF4A overexpression (Figure 6L,M). Collectively, these findings indicate that HNF4A promotes gastric cancer progression by transcriptionally activating F7, which subsequently regulates ITGA2 expression and downstream malignant phenotypes.

### 3.7 Identification and Characterization of Thonningianin A as a Potential F7‐Targeting Small‐Molecule Inhibitor

To identify small molecules targeting F7, we first examined the F7 structure (PDB ID: 5U6J) and performed computer‐aided virtual screening against the ligand‐binding pocket of human F7 corresponding to the binding site occupied by compound 14 (82J), involving key residues including Cys42–Cys58, Asp189, Ser195, Gly219, and Trp215. From a library of 48 572 compounds, virtual screening followed by filtering based on docking score, scaffold diversity, and molecular weight yielded 20 candidate compounds (Figure 7A and Table S4). Cytotoxicity assays in gastric cancer cells identified five compounds with inhibitory activity, including THA, platycodin D, 1,2,3,6‐tetragalloylglucose, chebulinic acid, and theaflavin 3,3′‐digallate (Figure S9A). Following compound treatment, western blot analysis showed that THA and platycodin D dose‐dependently reduced F7 protein expression (Figure S9B).

**FIGURE 7:**
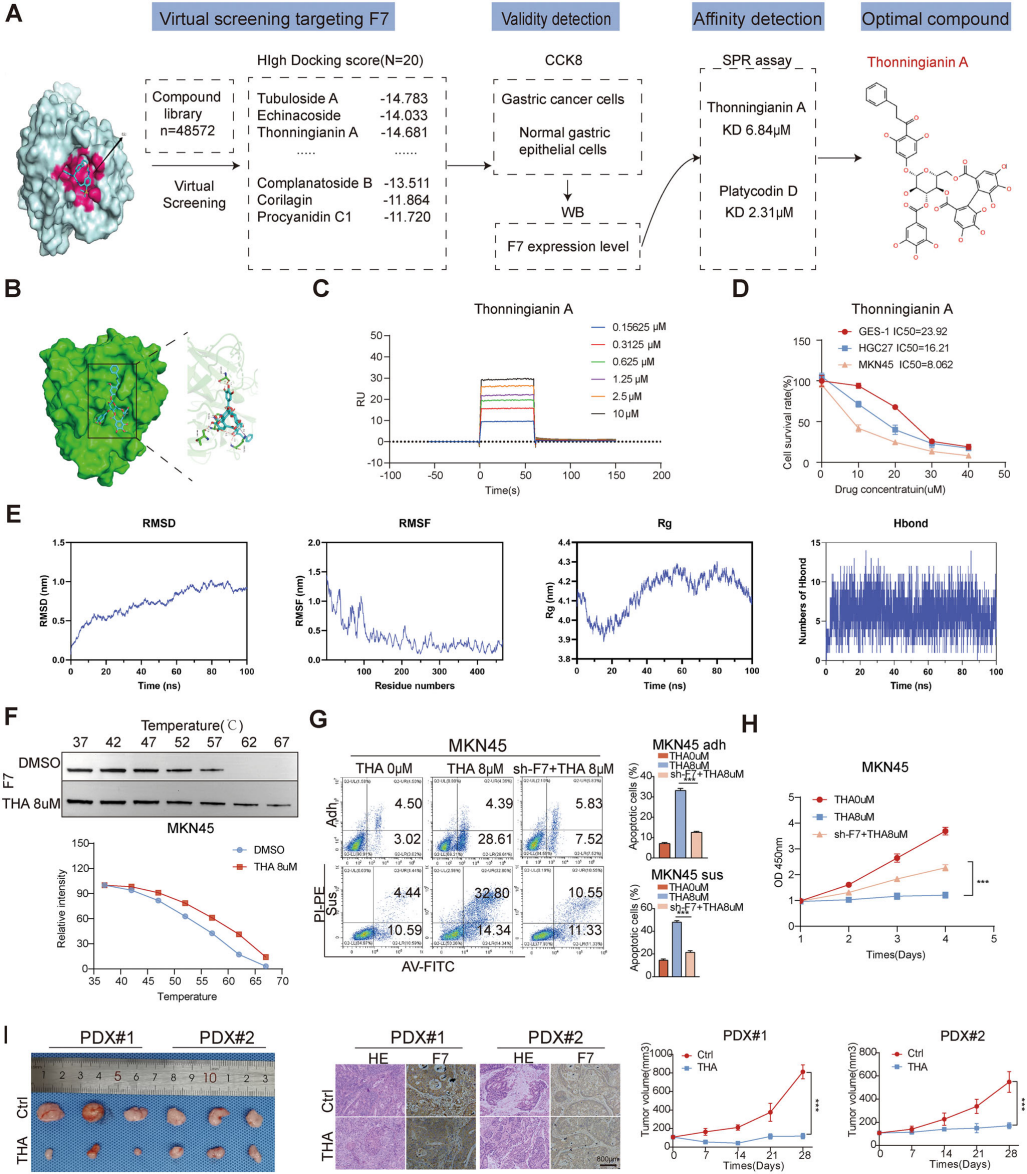
Screening and validation of ThonningianinA as a potential F7‐targeting small molecule in gastric cancer (A) Flowchart depicting the virtual screening and validation process used to identify candidate F7‐targeting compounds. (B) Molecular docking analysis showing the predicted binding mode and molecular interactions between THA and F7. (C) SPR sensorgram showing the binding affinity of THA to F7 at concentrations ranging from 0.15625 to 10 µM. (D) Dose–response curves showing the survival rates of GES‐1, HGC27, and MKN45 cells after THA treatment. (E) Molecular dynamics simulations the THA–F7 complex over 100 ns, including root‐mean‐square deviation (RMSD), root‐mean‐square fluctuation (RMSF), radius of gyration (Rg), and hydrogen‐bond analyses. (F) Cellular thermal shift assay (CETSA) showing that THA treatment increased the thermal stability of the F7 protein in MKN45 cells. (G,H) Apoptosis and proliferation assays showing that THA induced apoptosis and inhibited proliferation in MKN45 cells, whereas F7 knockdown attenuated these effects, suggesting that F7 contributes to THA‐mediated antitumor activity. (I) Gastric cancer PDX models showing reduced tumor growth following THA treatment. *p < 0.05; **p < 0.01; ***p < 0.001; ****p < 0.0001.

SPR confirmed direct binding of THA and platycodin D to F7, with dissociation constants (K_D_) of 6.84 and 2.31 µM, respectively (Figure 7C and Figure S9C). Half‐maximal inhibitory concentrations (IC_50_) were measured in normal gastric epithelial cells and gastric cancer cell lines. In high‐F7‐expressing MKN45 cells, THA exhibited an IC_50_ of 8.062 µM, which was lower than that of platycodin D (10.94 µM) (Figure 7D and Figure S9D). Therefore, THA was selected for further validation as a lead candidate F7‐targeting small‐molecule inhibitor. Molecular dynamics simulations over 100 ns supported a stable interaction between THA and F7. The complex RMSD fluctuated within 1.0 nm, RMSF values remained low across most regions except the protein N‐terminus, and the radius of gyration (R_g_) remained within 3.9–4.3 nm, indicating a compact and stable complex. Hydrogen‐bond analysis showed approximately 10 persistent hydrogen bonds, with a maximum of 14 hydrogen bonds, supporting a stable binding mode (Figure 7E).

To further validate the intracellular interaction between THA and F7, we performed a cellular thermal shift assay (CETSA). THA treatment markedly increased the thermal stability of F7 protein (Figure 7F), supporting intracellular target engagement between THA and F7. More importantly, when THA treatment was combined with F7 knockdown, the cytotoxic effect of THA and its inhibitory effect on anoikis resistance were significantly attenuated compared with those in cells without F7 knockdown (Figure 7G,H). These findings indicate that F7 is a functionally relevant target of THA and contributes to its antitumor activity.

To further assess the in vivo safety profile of THA, its toxicity was evaluated in NOD–SCID mice. THA was administered intraperitoneally at 0, 10, 20, or 40 mg kg^−1^ once daily for 14 days. Hematoxylin–eosin (H&E) staining of the heart, liver, spleen, lung, and kidney showed preserved tissue architecture at doses of 0–20 mg kg^−1^, whereas mild hepatic vacuolar changes were observed in the 40 mg kg^−1^ group (Figure S9E). Liver function analysis was abnormal in the 40 mg kg^−1^ group, including increased alanine aminotransferase (ALT) and aspartate aminotransferase (AST) levels, markedly elevated total bile acid (TBA), and increased direct bilirubin (DBIL) and total bilirubin (TBIL) levels. In contrast, renal function indices, including blood urea nitrogen (BUN) and creatinine (CREA), were not significantly altered, and plasma fibrinogen (FIB) levels changed only minimally (Figure S9F). Taken together, these findings indicate that THA is well tolerated at doses of 0–20 mg kg^−1^, whereas signs of hepatotoxicity were observed at doses ≥40 mg kg^−1^. To assess the antitumor efficacy of THA, gastric cancer PDX models were established in NOD–SCID mice. When tumors reached approximately 100 mm^3^, THA was administered at 20 mg kg^−1^. THA suppressed PDX tumor growth, with a stronger antitumor effect observed in high‐F7‐expressing PDX models (Figure 7I). Collectively, these results identify THA as a potential F7‐targeting small‐molecule inhibitor that suppresses gastric cancer growth, at least partly through inhibition of F7‐associated functions.

### 3.8 THA Enhances Anti–PD‐1 Therapy by Suppressing the F7‐Associated PD‐L1 Axis and Promoting CD8^+^ T‐Cell Immunity in Gastric Cancer Models

To assess the therapeutic effects of THA combined with immune checkpoint blockade, we established an MFC liver metastasis (MFC‐LM) model by injecting MFC cells into 615 mice. Treatment was initiated on day 7 and continued for the indicated duration (Figure 8A). In vivo imaging showed that both THA and anti–PD‐1 monotherapy gradually reduced metastatic signals compared with controls. Combination therapy resulted in the strongest inhibition of metastatic progression with the fewest detectable lesions (Figure 8B). At the experimental endpoint, gross examination and H&E staining of liver tissues confirmed the imaging results. Control mice exhibited extensive metastatic nodules, whereas both monotherapies partially reduced tumor burden. In contrast, combination therapy markedly decreased metastatic burden and preserved hepatic architecture (Figure 8C,D). Similarly, survival analysis demonstrated prolonged survival in the combination therapy group (Figure 8F). Flow cytometric analysis of tumor‐infiltrating immune cells in the MFC‐LM model revealed that both THA and anti–PD‐1 increased intratumoral CD3^+^CD8^+^ T cells and GZMB expression, with further enhancement observed in the combination group (Figure 8E). To determine whether THA regulates the F7‐associated PD‐L1 pathway in vivo, we examined F7 and PD‐L1 expression in tumor tissues following THA treatment. THA treatment markedly reduced PD‐L1 expression in tumor tissues, accompanied by decreased F7 expression, suggesting that THA inhibits the F7‐associated PD‐L1 regulatory axis in vivo (Figure 8G). Taken together, these findings highlight the immunomodulatory activity of THA through inhibition of the F7‐associated PD‐L1 regulatory axis and enhancement of CD8^+^ T‐cell immunity. To further validate these findings, we generated immune‐reconstituted gastric cancer PDX models in NOD–SCID mice (Figure 8H) and applied the same dosing regimen as that used in the MFC‐LM model. In two independent PDX models (PDX #1, PDX #2), gross examination at the experimental endpoint showed that THA and anti–PD‐1 monotherapy reduced tumor volume, whereas combination therapy resulted in the smallest tumors (Figure 8I). Tumor growth curves demonstrated that both monotherapies inhibited tumor growth, whereas combination therapy produced a stronger and more sustained inhibitory effect, resulting in significantly smaller tumors compared with controls and either monotherapy group (Figure 8I). IF analysis showed limited CD8^+^ T‐cell infiltration in control tumors, increased infiltration after either monotherapy, and further enrichment following combination therapy (Figure 8J).

**FIGURE 8:**
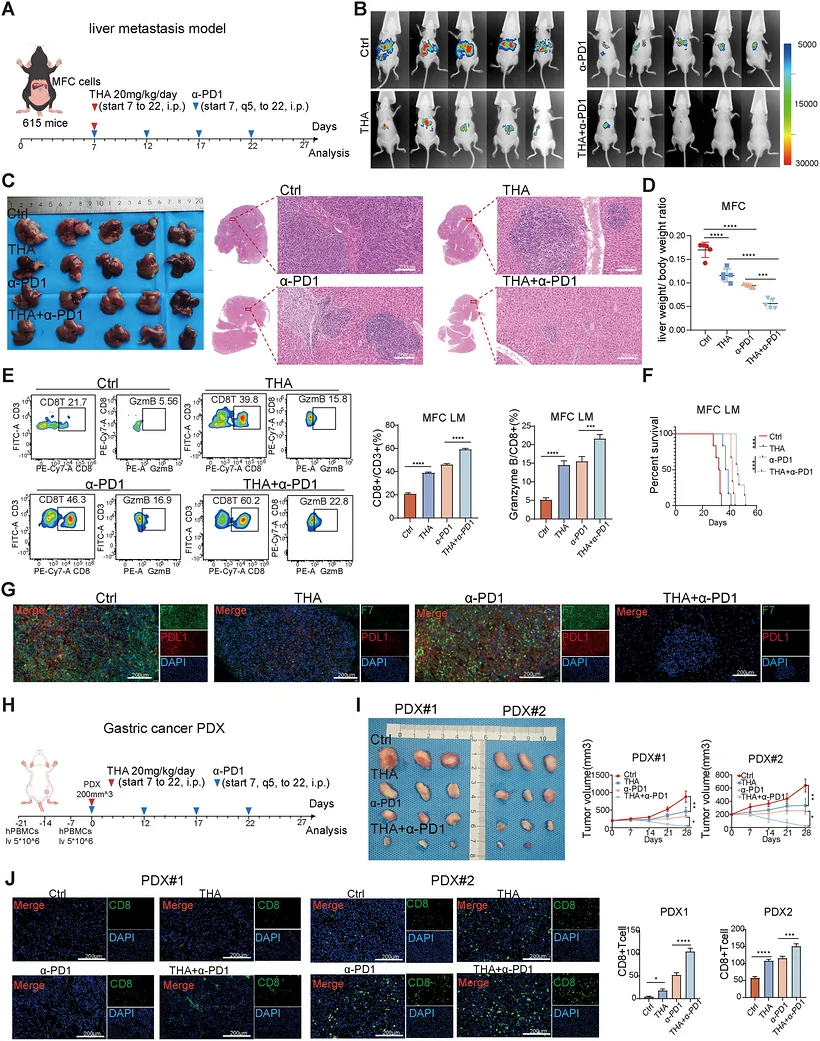
ThonningianinA enhances anti–PD‐1 therapy in liver metastasis and gastric cancer PDX models (A) Experimental design of MFC liver metastasis model treated with THA and an anti–PD‐1 antibody. (B,C) Bioluminescence imaging, gross liver images, and H&E staining showing reduced metastatic burden following THA, anti–PD‐1, or combination therapy, with the greatest inhibition observed in the combination group. (D) Liver weight/body weight ratio showing a significant reduction in metastatic burden in the THA plus anti–PD‐1 group compared with the respective monotherapy groups. (E) Flow cytometric analysis of CD8^+^ T cells and granzyme B expression in liver metastatic lesions. (F) Kaplan–Meier survival curves showing improved survival in mice receiving combination treatment. (G) Immunofluorescence staining of F7 and PD‐L1 in liver metastatic lesions, showing reduced F7 and PD‐L1 expression following combination therapy. (H) Schematic illustration of the gastric cancer PDX model and treatment strategy. (I) Tumor images and growth curves showing that THA plus anti–PD‐1 treatment reduced tumor growth in gastric cancer PDX models. (J) Immunofluorescence staining of CD8^+^ T cells in PDX tumors, showing increased CD8^+^ T‐cell infiltration following combination treatment. *p < 0.05; **p < 0.01; ***p < 0.001; ****p < 0.0001.

Collectively, THA combined with PD‐1 blockade enhanced antitumor activity by promoting CD8^+^ T‐cell infiltration and suppressing tumor growth in both syngeneic MFC‐LM and immune‐reconstituted gastric cancer PDX models.

## 4 Discussion

This study systematically elucidates the tumor‐promoting and immunomodulatory roles of the coagulation factor family member F7 in gastric cancer and proposes a therapeutic strategy targeting F7. We demonstrate that F7 is markedly upregulated in gastric cancer and is associated with aggressive clinicopathological characteristics and unfavorable outcomes. Previous studies have primarily focused on the effects of the TF–FVIIa complex on tumor biology, showing that this complex triggers PAR signaling, receptor tyrosine kinase pathways, and integrin pathways to promote tumor progression, angiogenesis, metastasis, and stemness maintenance [30, 31]. These findings are consistent with our previous observations that F7 promotes invasive phenotypes and anoikis resistance in gastric cancer [19]. However, emerging evidence suggests that coagulation factors may exert biological functions beyond the canonical coagulation cascade. For example, TF has been shown to promote immune evasion in triple‐negative breast cancer by reducing T‐cell infiltration [32] and to suppress cGAS–STING‐mediated innate immune surveillance through TREX1 stabilization in pancreatic ductal adenocarcinoma [10]. In addition, the association between tumor‐derived F7 and poor prognosis in breast cancer suggests that F7 may represent a conserved tumor‐promoting factor across different cancer types [13], providing indirect support for its pathogenic role in gastric cancer

Functionally, we demonstrated that tumor‐derived F7 enhances anoikis resistance and promotes migration behaviors of gastric cancer cells through an autocrine mechanism. Supplementation with rF7 restored these phenotypes following F7 knockdown, supporting the functional relevance of extracellular F7 signaling. In vivo, stable F7 knockdown markedly reduced metastatic burden and increased apoptosis within metastatic lesions.

Previous studies have long recognized the interplay between the coagulation–inflammation–immunity network and cancer [33, 34, 35]. In TCGA data and tissue cohorts assessed by IHC and mIF, high F7 expression was associated with reduced CD8^+^ T‐cell infiltration. In vitro PBMC co‐culture assays and immunocompetent mouse models further demonstrated that F7 knockdown enhanced CD8^+^ T‐cell infiltration and cytotoxicity while increasing the proportion of GZMB^+^ CD8^+^ T cells. Collectively, these findings support a role for F7 in shaping an immunosuppressive tumor microenvironment and impairing antitumor immunity.

In mechanistic investigations, we identified an extracellular interaction between F7 and ITGA2 at the tumor cell membrane. This interaction activates the downstream p‐FAK–NF‐κB signal reinforces ITGA2 expression through a positive‐feedback mechanism. In addition, ITGA2‐mediated PD‐L1 regulation and collagen I deposition within the ECM may contribute to reduced CD8^+^ T‐cell infiltration and cytotoxic activity. Prior studies show that FAK activation promotes NF‐κB nuclear translocation and transcriptional activity [36, 37, 38], whereas NF‐κB transcriptionally regulates ITGA2 expression, supporting the potential existence of an F7–ITGA2–FAK–NF‐κB regulatory circuit. Moreover, the ITGA2‐mediated PI3K–AKT pathway partly contributes to PD‐L1 upregulation and ECM remodeling, which may facilitate CD8^+^ T‐cell exclusion and functional impairment. Notably, multiple reviews and studies have systematically summarized the regulatory pathways governing PD‐L1 expression [26, 39], in which the PI3K–AKT and NF‐κB pathways upregulate PD‐L1 across multiple tumor types. To the best of our knowledge, this study identifies F7 as a previously unrecognized extracellular regulator of ITGA2, providing new insights into coagulation factor‐mediated tumor progression. Beyond tumor cell–intrinsic mechanisms, ECM remodeling represents an additional process associated with F7‐mediated immune evasion. We observed that F7 knockdown markedly reduced collagen deposition in metastatic lesions and was associated with increased CD8^+^ T‐cell infiltration. Previous studies have shown that dense or aligned collagen fibers and increased ECM stiffness are associated with impaired T‐cell migration [29]. Accordingly, reducing collagen crosslinking can overcome resistance to PD‐1/PD‐L1 blockade and improve therapeutic efficacy [40]. Our findings are consistent with these observations, suggesting that F7 may contribute to an immune‐excluded microenvironment through complementary regulation of PD‐L1 expression and ECM remodeling. Furthermore, we found that the transcription factor HNF4A directly binds to the F7 promoter and promotes F7 transcription. Notably, under suspension conditions, HNF4A undergoes nuclear translocation and further amplifies F7 expression, suggesting a stress‐responsive mechanism regulating F7 expression during metastatic adaptation. These results provide a mechanistic explanation for the upregulation of F7 under anoikis stress and further support the HNF4A–F7 axis as an upstream regulator of metastatic progression.

From a translational perspective, we identified the natural polyphenol THA as a potential F7‐targeting inhibitor. THA bound to F7 and increased F7 thermal stability in cells, supporting intracellular target engagement. THA exhibited preferential antitumor activity in F7‐expressing cells under anoikis stress and inhibited PDX tumor growth in vivo at a tolerable dose of ≤20 mg kg^−1^, with stronger effects observed in F7‐high models. When THA treatment was combined with F7 knockdown, its cytotoxic effect and inhibition of anoikis resistance were markedly attenuated, indicating that F7 is a functionally relevant target contributing to THA‐mediated antitumor activity. Although these findings provide functional evidence supporting THA as an F7‐targeting compound, future studies using mutagenesis of predicted binding residues will be required to further define the molecular determinants underlying the THA–F7 interaction and its specificity.

THA treatment reduced PD‐L1 expression in tumor tissues, accompanied by decreased F7 expression, suggesting inhibition of the F7‐associated PD‐L1 regulatory axis in vivo. Together with our findings that F7 contributes to immune evasion partly through impaired CD8^+^ T‐cell–mediated antitumor immunity, these results suggest that THA may alleviate F7‐mediated immunosuppression. Consistently, THA combined with anti–PD‐1 therapy showed enhanced antitumor activity in a mouse liver metastasis model and immune‐reconstituted gastric cancer PDX models, accompanied by increased CD8^+^ T‐cell infiltration and elevated GZMB expression. These findings support a therapeutic strategy in which F7 inhibition may alleviate tumor‐associated immunosuppression, thereby enhancing responsiveness to immune checkpoint blockade.

For clinical translation, the expression levels of F7, HNF4A, ITGA2, and PD‐L1, together with collagen‐related features, may serve as potential biomarkers for patient stratification and therapeutic response assessment. Early‐phase clinical studies may prioritize patients with metastatic or immunotherapy‐refractory gastric cancer characterized by high F7 expression or immune‐cold tumor microenvironments. Nevertheless, residue‐level mutagenesis of the predicted THA‐binding pocket and CD8^+^ T‐cell depletion following THA plus anti–PD‐1 treatment were not performed in this study. Therefore, potential F7‐independent or off‐target effects cannot be completely excluded, and future studies incorporating binding‐site mutagenesis, F7 rescue experiments, and CD8^+^ T‐cell depletion during combination therapy are warranted.

Despite the integration of multi‐omics analyses, patient cohorts, and multiple experimental models several limitations remain. First, although the IHC cohort included a relatively large sample size, multicenter prospective studies with standardized clinical treatment protocols are needed to validate the clinical relevance of F7. Second, although our findings demonstrate that F7 promotes collagen deposition and is associated with reduced CD8^+^ T‐cell infiltration, additional collagen‐targeted intervention studies are required to determine whether ECM remodeling is directly responsible for immune exclusion. Third, the biological context of extracellular F7 signaling, including its relationship with TF and other components of the coagulation system, platelets, and endothelial cells, requires further investigation. Fourth, systemic targeting of F7 may require careful evaluation of potential coagulation‐related safety risks. Future studies should optimize THA selectivity, administration strategies, pharmacokinetic and toxicological profiles while defining the safety parameters for combination with anticoagulant or antiplatelet therapies.

In summary, our study demonstrates that HNF4A transcriptionally activates F7 and promotes F7 expression and autocrine signaling. Secreted F7 engages ITGA2 to activate FAK–NF‐κB signaling, which in turn induces ITGA2 transcription and cell‐surface enrichment. This signaling axis contributes to ECM remodeling and regulates cellular adhesion and motility, thereby facilitating lymph node and hepatic metastasis. In addition, through ITGA2‐associated PD‐L1 regulation, this axis may contribute to an immunosuppressive microenvironment characterized by reduced CD8^+^ T‐cell infiltration and cytotoxic activity. Under anoikis stress, HNF4A undergoes nuclear translocation to enhance F7 transcription, thereby reinforcing anoikis resistance and metastatic potential. Therapeutically, F7 inhibition attenuates metastatic capacity and immunosuppression and enhances the antitumor efficacy of anti–PD‐1 therapy (Figure 9). These findings suggest that an inhibitory strategy targeting F7, particularly in combination with PD‐1 blockade, may benefit patients with high F7 and/or ITGA2 expression or immune‐excluded gastric tumors, pending future clinical validation.

**FIGURE 9:**
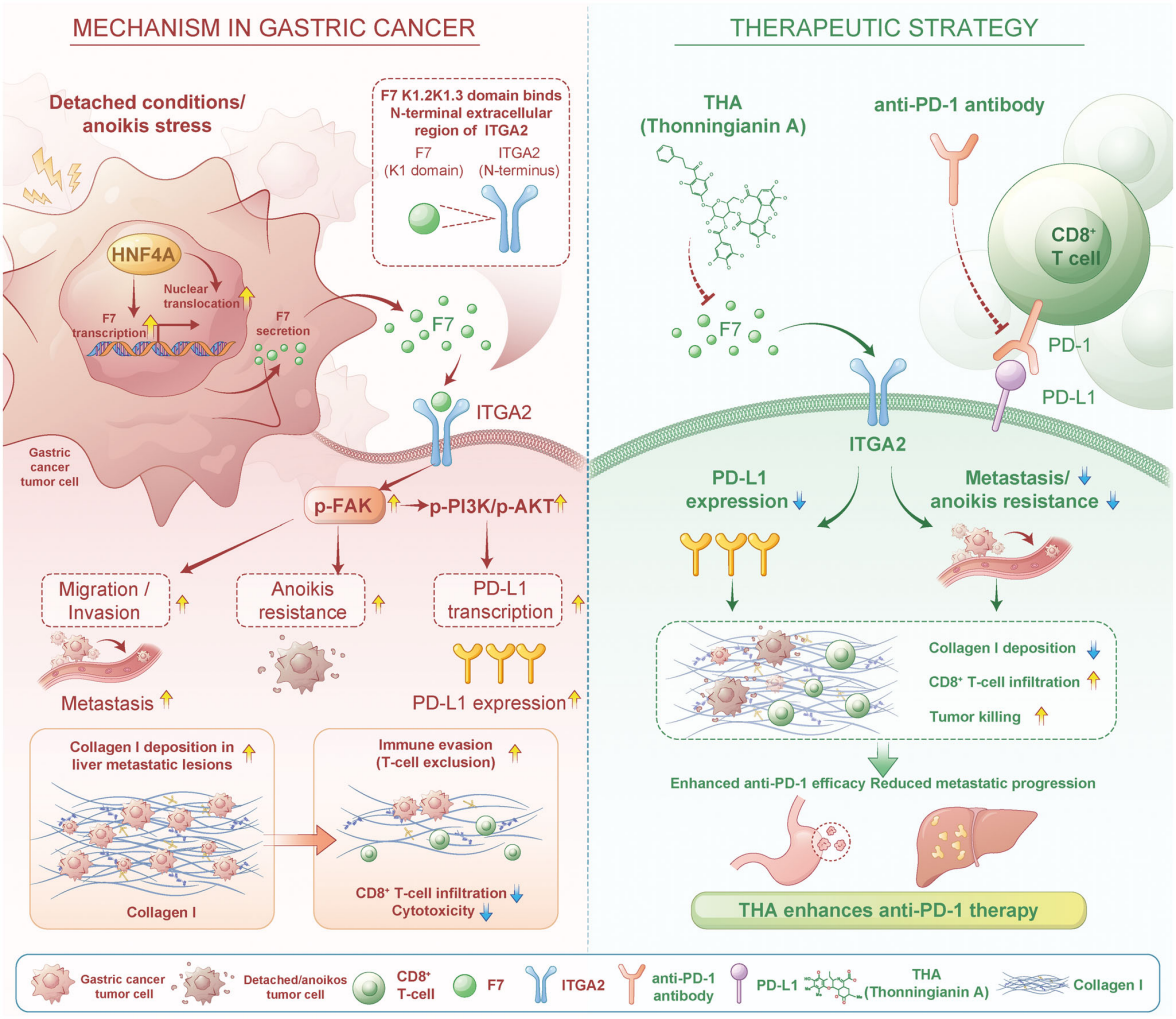
Schematic illustration of the proposed mechanism by which F7 promotes gastric cancer metastasis and immune evasion.

## 5 Conclusions

In conclusion, our study identifies F7 as a critical driver of gastric cancer metastasis and immune evasion by linking anoikis resistance with tumor microenvironment remodeling. Mechanistically, F7 promotes malignant progression through ITGA2‐mediated signaling and suppresses antitumor immunity by regulating PD‐L1 expression and CD8^+^ T‐cell dysfunction. HNF4A‐mediated transcriptional activation of F7 under anoikis stress further reveals a mechanism underlying F7 upregulation during metastasis. Importantly, targeting F7 with THA enhances the efficacy of PD‐1 blockade by restoring CD8^+^ T‐cell–mediated antitumor immunity. These findings establish F7 as a potential therapeutic target and provide a rationale for combining F7 inhibition with immune checkpoint blockade in metastatic gastric cancer.

## Author Contributions

**Lei Gao** and **Qinying Han** contributed equally. **Lei Gao**, **Qinying Han**, **Bofang Wang**, **Ying Zhang**, and **Hao Chen**: conceptualization, original draft, methodology, review and editing, **Hao Chen**: funding acquisition, and supervision. **Lei Gao**, **Qinying Han**, **Bofang Wang**: carried out the experiments and generated the figures. **Lei Gao** wrote the paper. **Xuemei Li**, **Baohong Gu**, **Yunpeng Wang**, **Weidong Li**, **Lin Xiang**: participated in part of the experiments.

## Funding

This research was supported by National Natural Science Foundation of China (82473266 and 82160129), Key Project of Science and Technology in Gansu province (22ZD6FA054), Major scientific research project for scientific and technological innovation in the health industry of Gansu Province (GSWSZD‐2024‐17), General Project of Gansu Province Joint Research Fund (24JRRA929), Key Talents Project of Gansu Province (2019RCXM020), Gansu Province Postgraduate, Innovation Star Program (2025CXZX‐115), and Cuiying Scientific and Technological Innovation Program of Lanzhou University Second Hospital (CY2023‐ZD‐01) to Hao Chen. National Natural Science Foundation of China (82560488); Cuiying Scientific and Technological Innovation Program of Lanzhou University Second Hospital (CY2023‐QN‐A01); Gansu Province Science and Technology Plan Project (24JRRA373). The funder(s) had no role in the study design; collection, analysis, or interpretation of data; writing of the report; or the decision to submit the paper for publication. The corresponding author had full access to all the data in the study and had final responsibility for the decision to submit for publication.

## Ethics Statement

The human study protocol was reviewed and approved by the Ethics Committee of the Second Hospital of Lanzhou University (Ethical Approval No. 2023A‐603). Owing to the retrospective nature of the study and the use of anonymized data, the requirement for individual informed consent was waived by the ethics committee. All animal experiments were approved by the Animal Ethics Committee of the Second Hospital of Lanzhou University (Ethical Approval No. 2023A‐603) and were performed in accordance with institutional guidelines and national regulations on the care and use of laboratory animals. Patient consent for publication, Not required, as no identifiable personal data are presented, and the animal experiments did not involve human participants.

## Consent

This manuscript is original, has not been published previously, and is not under consideration by any other journal. All authors have approved the submission and agree with its submission to the Advanced Science.

## Conflicts of Interest

The authors declare no conflicts of interest.

## Supporting information




**Supporting File**: advs77095‐sup‐0001‐SuppMat.pdf.

## Data Availability

The original files of all proteome mass spectrometry analyses have been deposited in the ProteomeXchange database through iProX (www.iprox.org), with the identification number being IPX0015308000 (iProX), or ProteomeXchange(PXD073322). The RNA‐seq datasets generated during the current study are available in the NCBI SRA repository under BioProject accession PRJNA1405598.
