# Supplementary material for: F7 Drives Gastric Cancer Metastasis Through Anoikis Resistance and Tumor Microenvironment Remodeling
Source: Adv Sci (Weinh). 2026 Aug 17:e77095. Online ahead of print. doi: 10.1002/advs.77095 (PMC13479850; doi:10.1002/advs.77095)
Supplement: Supplementary file 1 [file ADVS-9999-e77095-s001.pdf]

Supplementary Materials for

F7 drives gastric cancer metastasis through anoikis resistance and tumor microenvironment remodeling

Correspondence to: ery_chenh@lzu.edu.cn； sj_chnh@sjtu.edu.cn(Hao Chen)

Materials and Methods

Patients and Specimens

This study included 214 paired gastric cancer and adjacent non-tumor tissue samples collected from patients who underwent gastrectomy at the Second Hospital of Lanzhou University between January 2017 and January 2022. All cases were pathologically confirmed as gastric cancer and were followed up after surgery. Immunohistochemical staining was performed on these samples to evaluate F7 expression; this set of 214 paired samples was defined as Cohort 2. In addition, 32 paired fresh postoperative gastric cancer and adjacent non-tumor tissue samples were collected for quantitative real-time polymerase chain reaction (qRT-PCR) analysis and were defined as Cohort 1. Among these samples, eight pairs of fresh tissue samples were further subjected to immunoblot analysis to assess F7 protein expression. Written informed consent was obtained from all patients. This study was approved by the Ethics Committee of the Second Hospital of Lanzhou University (Ethical Approval No. 2023A-603), and all sample collection and use were conducted in accordance with relevant ethical guidelines.

Animal Studies

All animal experiments were conducted in accordance with the "*Guidelines for the Care and Use of Laboratory Animals*" and were approved by the Ethics Committee of the Second Hospital of Lanzhou University (Ethical Approval No. 2023A-412). NOD–SCID mice were purchased from Jiangsu Jicui Yaokang Biotechnology Co, Ltd, and 615 mice were obtained from the Institute of Hematology, Chinese Academy of Medical Sciences Tianjin, China..

Liver metastasis models were established by splenic injection, whereas lymph node metastasis models were established by footpad injection. For the liver metastasis model, 2×10^6 MKN45 cells in the logarithmic growth phase were injected into the spleens of 6-week-old female NOD–SCID mice weighing 20 ± 1 g. Mice were allocated to the following groups: sh-Ctrl (n = 5) and sh-F7 (n = 5), or sh-Ctrl (n = 5) and sh-HNF4A (n = 5). Mice were sacrificed 4 weeks after tumor cell inoculation. For the lymph node metastasis model, 1×10^6 MKN45 cells in the logarithmic growth phase were injected into the footpads of 6-week-old female NOD–SCID mice weighing 20 ± 1 g, with the same group allocation as described above.

To evaluate the effect of F7 on antitumor immune responses, 1×10^6 MFC cells in the logarithmic growth phase were injected into the spleens of 6-week-old male 615 mice weighing 22 ± 1 g to establish a liver metastasis model. Mice were divided into sh-Ctrl (n = 5) and sh-F7 (n = 5) groups. To assess the therapeutic effect of THA combined with anti–PD-1 treatment, 6-week-old male 615 mice were assigned to four groups: Ctrl (n = 5), THA (n = 5), anti–PD-1 (n = 5), and THA + anti–PD-1 (n = 5). Mice were sacrificed 4 weeks after the start of the experiment.

For the PDX model, 6-week-old female NOD–SCID mice weighing 20 ± 1 g were divided into four groups: Ctrl (n = 5), THA (n = 5), anti–PD-1 (n = 5), and THA + anti–PD-1 (n = 5). Mice

were sacrificed 4 weeks after the start of treatment. All mice were euthanized by anesthetic overdose.

At the experimental endpoint, tumor samples were collected and subjected to hematoxylin and eosin (H&E) staining, immunohistochemistry, immunofluorescence, and flow cytometry. All experiments were randomized according to animal identification numbers. Investigators who administered treatments were aware of the group allocation at different stages of the experiment. Statistical analyses were performed using a two-tailed Student's *t*-test for comparisons between two groups and one-way ANOVA followed by post hoc testing for comparisons among three or more groups.

Virtual Screening

The three-dimensional structure of human F7 protein has been resolved and is available in the Protein Data Bank (PDB ID: 5U6J). Therefore, virtual screening was performed to identify compounds targeting the binding pocket of human F7, which contains key amino acid residues including Cys42, Cys58, Asp189, Ser195, Gly219, and Trp215. Virtual screening was conducted using Schrödinger Maestro 12.8, and three-dimensional visualization was performed using PyMOL. Compounds from the HY-L001P, HY-L025, HY-L031, HY-L021, and HY-L0107V libraries were screened. The two-dimensional structures of these compounds were processed using the LigPrep module in Schrödinger, including hydrogen atom addition and energy minimization. The resulting three-dimensional structures were then subjected to virtual screening. Prepared compounds were imported using the Virtual Screening Workflow module, and molecular docking was performed using the Glide module.

Cell Lines and Cell Culture

The gastric cancer cell lines MKN45 and HGC27, normal gastric epithelial cells GES-1, and mouse-derived gastric cancer cell line MFC were obtained from the Gansu Provincial Key Laboratory of Environmental Tumor. HEK293T cells were purchased from Wuhan Zishan Biotechnology Co., Ltd. All cell lines were routinely authenticated, and no mycoplasma contamination was detected. Gastric cancer cell lines were cultured in RPMI 1640 medium (Gibco, 8117072) supplemented with 10% fetal bovine serum (ExCELL, FSD500), whereas HEK 293T cells were cultured in DMEM medium (Gibco, 8121713) supplemented with 10% fetal bovine serum (ExCELL, FSD500). All cells were maintained at 37 °C in a humidified incubator containing 5% CO_2. T cells were cultured in RPMI 1640 medium supplemented with 10% fetal bovine serum (ExCELL, FSD500), 1% penicillin/streptomycin, 5 μg/mL CD3ε (BioLegend, #100340), 1 μg/mL CD28 (BioLegend, #302934), and 20 ng/mL mIL-2 (R&D Systems, #402-ML) under the same culture conditions.

Generation of Anoikis-Resistant Cells

Anoikis-resistant sublines were generated from the gastric cancer cell lines MKN45 and HGC27 using a cyclic suspension selection protocol. Briefly, exponentially growing cells were dissociated with 0.25% trypsin–EDTA to obtain single-cell suspensions, resuspended in complete medium, and seeded into ultra-low attachment plates to prevent adhesion. Cells were maintained under suspension conditions at 37 °C in a humidified incubator containing 5% CO_2 for 48 h to induce detachment stress. Thereafter, the entire suspension, including cell aggregates, was collected, centrifuged, and replated onto standard tissue culture-treated dishes to allow reattachment. After 12–24 h, the supernatant containing nonadherent or dead cells was discarded, and cultures were gently washed with PBS. Only re-adherent viable cells were retained and expanded. This 48 h suspension–readhesion cycle was repeated five times to enrich anoikis-tolerant populations. The resulting stable sublines were designated MKN45-AR and HGC27-AR,

and the corresponding parental cells were maintained in parallel as controls for subsequent experiments.

Immunohistochemistry

Immunohistochemical staining and evaluation were performed according to standard pathological procedures. Samples were fixed in 4% paraformaldehyde, embedded, and stored until use. After deparaffinization, immunohistochemical staining was performed using the MaiXin Biotechnology immunohistochemistry kit (KIT-9730), and color development was performed using the DAB method (KIT-0016). Sections were counterstained with hematoxylin for nuclear visualization, blued, dehydrated, cleared, and mounted. Images were then acquired and analyzed. Detailed information on antibody suppliers and concentrations is provided in Table S2.

RT-qPCR

Reverse transcription quantitative PCR (RT-qPCR) was used to assess transcript levels. RNA concentration and purity were evaluated using a spectrophotometer. cDNA was synthesized from total RNA, and reverse transcription was performed using the SweScript All-in-One Blue RT SuperMix for qPCR kit (Servicebio, G3329-100). Amplification was carried out using the SweScript One-Step RT-qPCR Kit (Servicebio, G3335-50). Expression levels were normalized to verified reference genes, and relative abundance was calculated using the $2^{-\Delta\Delta Ct}$ method. The specific primer sequences are listed in the Supplementary Table 1.

Western Blot

Protein levels were assessed by western blot analysis. Briefly, samples were treated with denaturing loading buffer, and proteins were separated by SDS-PAGE and transferred onto PVDF membranes. After blocking, membranes were incubated with primary antibodies against the target proteins, followed by species-matched secondary antibodies. Chemiluminescent substrates were used for detection, and signals were captured using a digital imaging system. Band intensities were quantified using ImageJ normalized to housekeeping proteins or total protein staining. Detailed information on antibody suppliers and concentrations is provided in Table S2.

Lentivirus-Transfected Stable Cell Lines

The F7 knockdown lentivirus was purchased from Shanghai GeneChem Co., Ltd, and the viral sequences are as follows: F7-RNAi(95729-1) CACCCACAGTTGAATATCCAT, F7-RNAi(95730-1) CAGGTCCTGTTGTTGGTGAAT, F7-RNAi(95731-1) TGCCTCAAGTCCATGCCAGAA, with the control sequence TTCTCCGAACGTGTCACGT.The HNF4A lentivirus was purchased from Shanghai GenePharma Co., Ltd., and the viral sequences are as follows: HNF4A-RNAi(198) GCCTACACCACCCTGGAATTT, HNF4A-RNAi(408) CACATGTACTCCTGCAGATTT, HNF4A-RNAi(511) AGAAGGAAGCCGTCCAGAATG, with the control sequence TTCTCCGAACGTGTCACGT. The lentivirus for overexpressing F7 and HNF4A was also purchased from Shanghai GenePharma Co., Ltd. To establish stable knockdown and overexpression cell lines, cells were selected by adding puromycin 48h after lentiviral transfection.

Flow Cytometry

For flow cytometric analysis, adherent cells were collected after digestion with trypsin without EDTA, whereas suspension cells were collected directly by centrifugation. After washing, cells were stained in a 100 μL reaction volume. The following kits were used: Annexin V-FITC/PI Cell Apoptosis Detection Kit (Servicebio, G1511-50T) and Annexin V-APC/7-AAD Apoptosis Kit (MULTI SCIENCES, AP105). For tumor tissues, freshly collected samples were minced into small pieces, mechanically dissociated through a 200-mesh screen, and filtered through a 70 μm cell strainer. Red blood cells were lysed using Red Blood Cell Lysis Buffer (Servicebio, G2015-500ML). The single-cell suspension was resuspended in 100 μL of pre-chilled live-cell staining

solution, consisting of 0.5 μL FVS per sample diluted in PBS, and incubated on ice for 10 min. Surface staining was performed by incubation with a surface antibody mixture for 30 min. For intracellular cytokine staining, cells were stimulated for 3–6 h, treated with 0.1% Triton, and then stained. Cells were then washed, resuspended, and stored at 4 °C until analysis. Flow cytometric analysis was performed using a CytoFLEX flow cytometer. Flow cytometry data were analyzed using FlowJo software. Antibody details and supplier information are provided in Table S2.

Co-Immunoprecipitation

For exogenous co-immunoprecipitation (Co-IP), HEK293T cells were transfected with the indicated plasmids according to the experimental groups and incubated for 48 h. Cellular proteins were extracted using Pierce™ IP Lysis Buffer (Thermo Scientific Pierce, 87787). For HA-tagged proteins, lysates were incubated with Anti-HA Magnetic Beads (MCE, HY-K0201). For FLAG-tagged proteins, lysates were incubated with Anti-FLAG Magnetic Beads (MCE, HY-K0207) overnight at 4 °C. After magnetic separation using a magnetic rack, the supernatant was discarded, and the beads were washed. Protein complexes were eluted under denaturing conditions for subsequent detection. For endogenous Co-IP, the BeaverBeads™ Protein A or Protein A/G Immunoprecipitation Kit was used.

ChIP and Dual-Luciferase Reporter Assays

All plasmids were purchased from Guangzhou Youming Biotechnology Co., Ltd. For transfection, cells were seeded and cultured to 70–90% confluence before plasmid transfection. The plasmid-to-Lipofectamine 2000 ratio was 2:1. After 6 h, the culture medium was replaced, and cells were collected for subsequent experiments 48 h after transfection. The dual-luciferase reporter assay was performed using the Dual-Luciferase® Reporter Assay System (Promega, E1910) according to the manufacturer's instructions. ChIP assays were performed using the SimpleChIP Enzymatic Chromatin IP Kit (Magnetic Beads) (Cell Signaling Technology, #9003). Detailed information on antibody suppliers and concentrations is provided in Table S2.

T-Cell Cytotoxicity Assays

For T-cell activation, 12-well plates were coated with CD3 (2 μg/mL in PBS) and CD28 (1 μg/mL in PBS). Human PBMCs were extracted and resuspended in RPMI 1640 medium supplemented with IL-2 (400 U/mL). The cell suspension (1 mL) was mixed gently and added to the coated 12-well plates. After 36h, the T cells were activated. The cells were further cultured and expanded for subsequent experiments. Tumor cells were labeled with CFSE (CellTrace™ CFSE, Invitrogen™ C34554), then co-cultured with T cells for 24h. Afterward, cells were collected for flow cytometric analysis of apoptosis.

$CD8^+$ T-Cell Depletion In Vivo (Mouse Model)

In the 615 MFC gastric cancer liver metastasis mouse model, $CD8^+$ T-cell depletion was performed by intraperitoneal injection of 100 μg anti-mouse CD8α antibody (Selleck Chemicals, Cat. No. A2102; Lot No. A210230; clone 2.43). The first injection was administered on day 2 after tumor inoculation, followed by a second injection on day 9 to maintain effective depletion. Depletion efficiency was verified by flow cytometric analysis of peripheral blood and immunofluorescence staining of liver metastatic tissues, enabling assessment of the role of $CD8^+$ T cells in F7-mediated tumor suppression.

IP–MS Analysis

MKN45 cell proteins were extracted using RIPA lysis buffer supplemented with protease and phosphatase inhibitors, and protein concentrations were determined using a BCA assay. Equal amounts of protein, typically 500 μg–1 mg, were used for co-immunoprecipitation (Co-IP) with the BeaverBeads™ Protein A Immunoprecipitation Kit according to the manufacturer's

instructions. Briefly, the target antibody or isotype control was incubated with magnetic beads overnight at 4 °C with gentle rotation. After incubation, the beads were washed 3–5 times with the provided wash buffer to remove nonspecific proteins. Bound proteins were then eluted using the elution buffer. The eluted proteins were reduced, alkylated, and digested with trypsin. The resulting peptides were desalted and concentrated before LC–MS/MS analysis. Mass spectrometry was performed by Megg Biotech Company. Input and IgG controls were included to validate the specificity of immunoprecipitation. All procedures were performed on ice or at 4 °C to minimize protein degradation, and care was taken to avoid bead loss during washing.

Surface Plasmon Resonance

Protein immobilization was performed using the amine coupling method. Briefly, the target protein was immobilized on the sensor chip surface, and interactions between the immobilized protein and small-molecule compounds were then measured. Each compound was diluted to a series of concentrations in a 96-well plate. Binding analysis was performed by flowing each compound over the target protein-coupled chip surface from the lowest to the highest concentration. The flow rate was set to 30 μL/min, with a contact time of 150 s. Data were collected using BIAcore T200 Control Software (version.2.0, GE Healthcare), and signals from the reference channel were subtracted. Binding and dissociation constants were obtained by globally fitting the data to a 1:1 Langmuir binding model using BIAcore T200 Evaluation Software (v.2.0, GE Healthcare).

Statistical Analyses

Statistical analyses were performed using GraphPad Prism software (version 9.0; GraphPad Inc., USA). Unless otherwise stated, all experiments were performed with three independent biological replicates. Data are presented as the mean ± standard deviation (SD). Comparisons between two groups were performed using a two-tailed unpaired Student's t-test, whereas comparisons among multiple groups were performed using one-way analysis of variance (ANOVA). A p-value < 0.05 was considered statistically significant. Detailed statistical methods are provided in the corresponding figure legends.

Supplementary Text

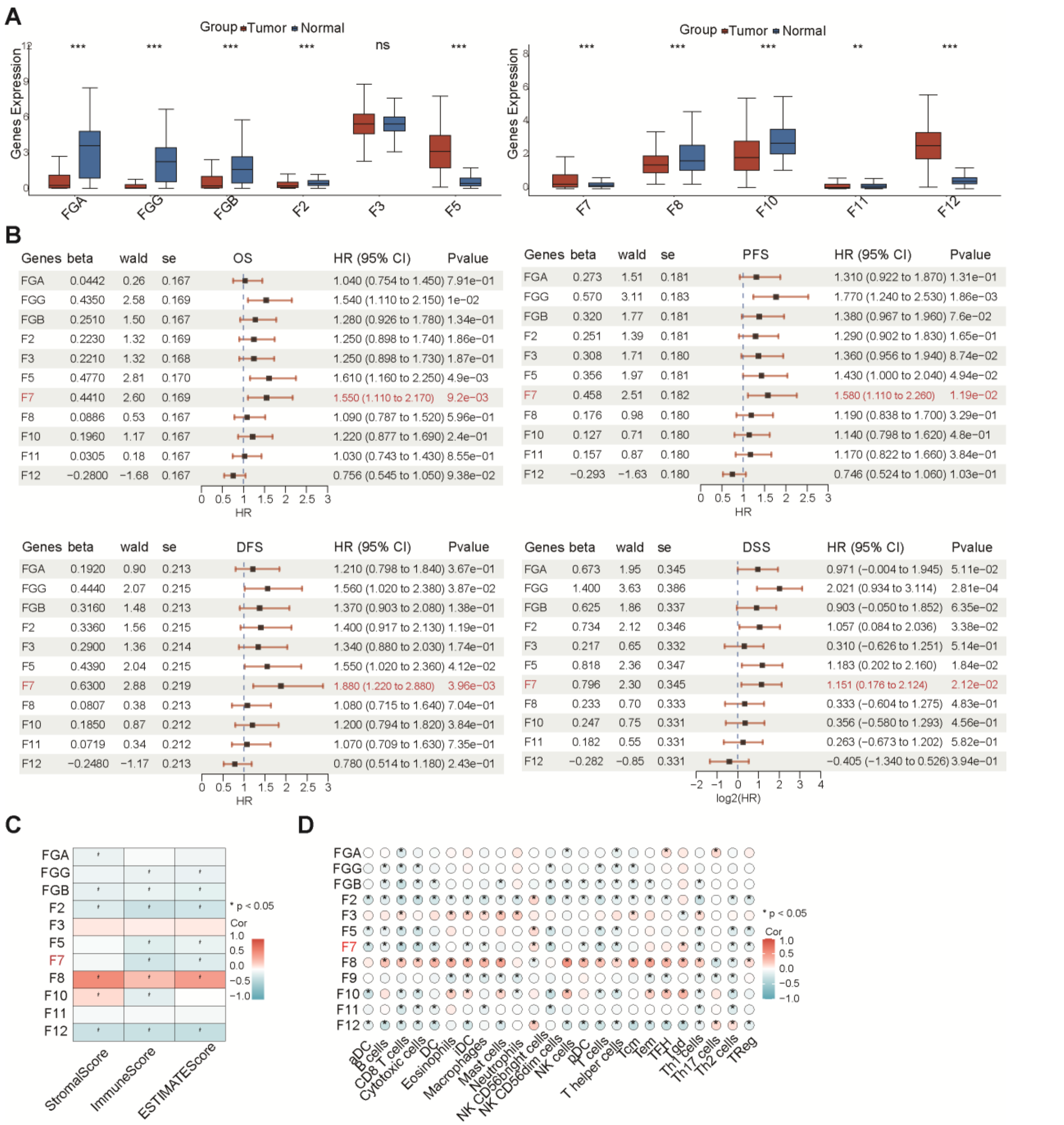

Figure S1. Coagulation factor genes are dysregulated in gastric cancer, and F7 is a consistent adverse prognostic marker. (A) Box plots showing the expression of fibrinogen chain genes (FGA, FGG, and FGB) and coagulation factor genes (F2, F3, F5, F7, F8, F10, F11, and F12) in tumor and normal tissues from a public cohort. (B) Univariate Cox regression analyses of overall survival (OS), progression-free survival (PFS), disease-free survival (DFS), and disease-specific survival (DSS). F7 showed the strongest and most consistent association with unfavorable survival outcomes across endpoints. (C) Heatmap showing correlations between each coagulation-related gene and ESTIMATE-derived scores, including StromalScore, ImmuneScore, and ESTIMATEScore. (D) Bubble plot showing correlations between each coagulation-related gene and immune-cell infiltration estimated by CIBERSORT. *$p < 0.05$; **$p < 0.01$; ***$p < 0.001$; ****$p < 0.0001$.

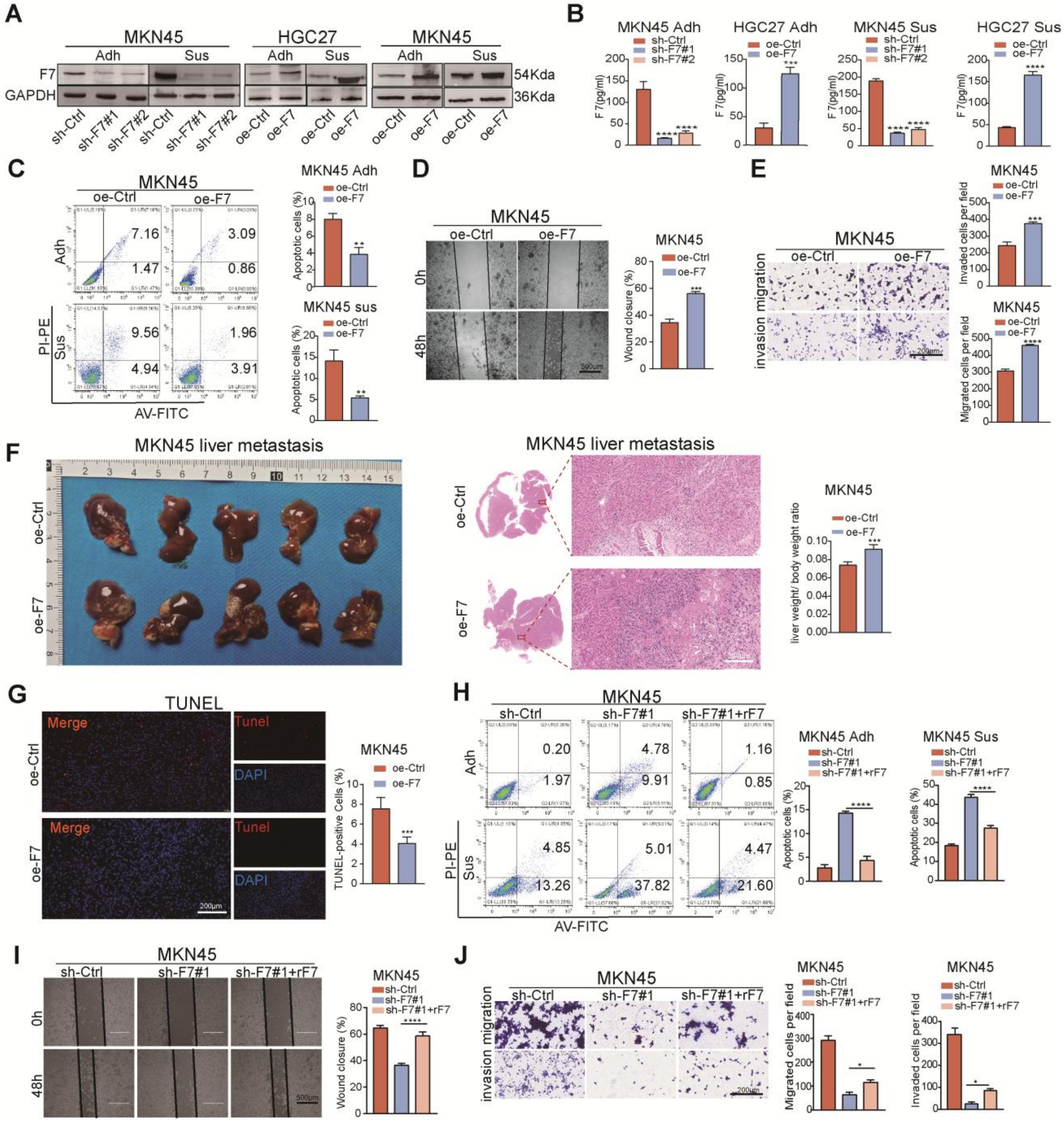

Figure S2. F7 promotes gastric cancer cell survival, migration, invasion, and liver metastasis. (A,B) Western blot and ELISA analyses confirming F7 knockdown (sh-F7#1/#2) in MKN45 cells and F7 overexpression (oe-F7) in HGC27 cells under adherent (Adh) and suspension (Sus) conditions. (C) Flow cytometric analysis showing that F7 overexpression reduced apoptosis in MKN45 cells. (D,E) Wound-healing and Transwell assays showing that F7 overexpression enhanced gastric cancer cell migration and invasion in MKN45 cells. (F) Liver metastasis model showing that F7 overexpression increased metastatic burden, as demonstrated by gross liver images and hematoxylin and eosin (H&E) staining. (G) TUNEL staining showing decreased apoptosis in liver metastatic lesions formed by F7-overexpressing cells. (H–J) Rescue experiments showing that

recombinant F7 (rF7) restored anoikis resistance, migration, and wound closure in F7-knockdown cells. *$p < 0.05$, **$p < 0.01$, ***$p < 0.001$, ****$p < 0.0001$.

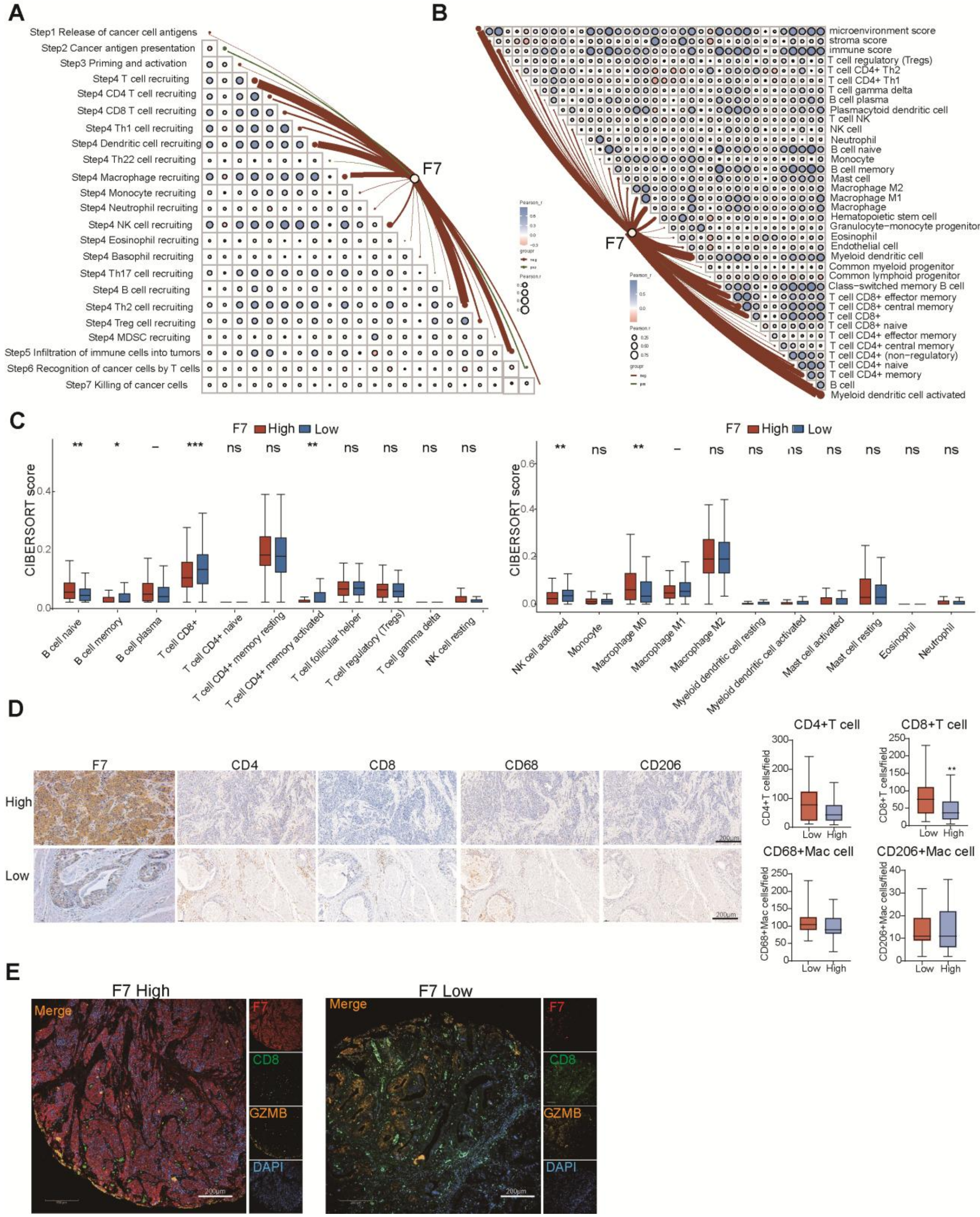

Figure S3. F7 expression is associated with immune cell infiltration and cytotoxic activation in gastric cancer. (A) Correlation analysis between F7 expression and different steps of the cancer-immunity cycle. (B) Correlation analysis between F7 expression and immune cell infiltration. (C) Box plots showing immune cell infiltration levels in tumors with high or low F7 expression. (D)

Representative immunohistochemical (IHC) staining images of F7, CD4, CD8, CD68, and CD206 in tumor tissues with high or low F7 expression. (E) Immunofluorescence staining showing the spatial association among F7, CD8, and granzyme B in tumor tissues with high or low F7 expression. *$p < 0.05$, **$p < 0.01$, ***$p < 0.001$, ****$p < 0.0001$.

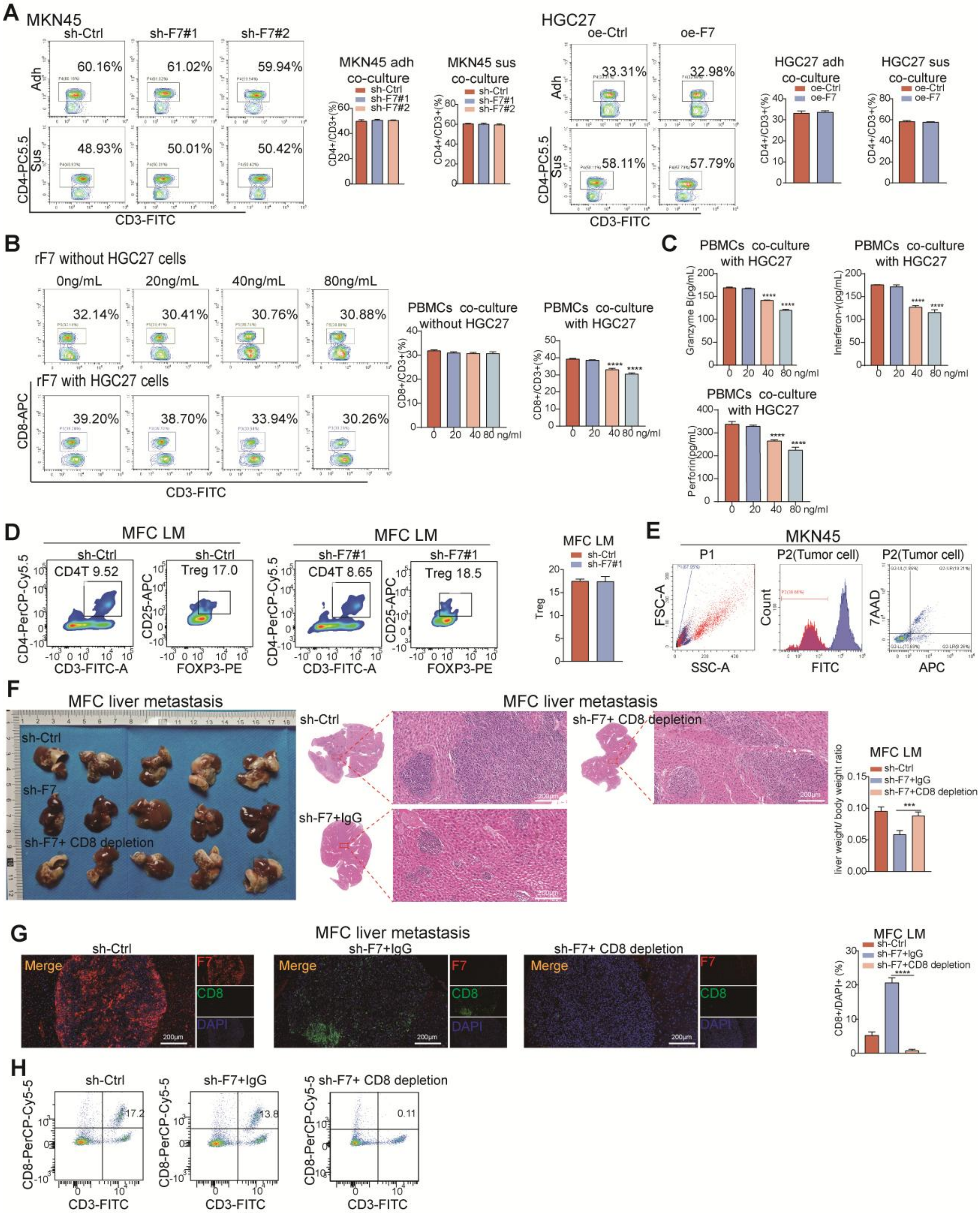

Figure S4. CD8⁺ T-cell depletion partially reverses the reduction in liver metastasis induced by F7 knockdown. (A) Flow cytometric analysis of CD4⁺ T-cell populations in PBMCs co-cultured with MKN45 or HGC27 cells. The corresponding quantification is shown on the right. (B) Flow cytometric analysis of CD8⁺ T-cell populations (CD3⁺CD8⁺) in PBMCs cultured with or without

HGC27 cells and treated with various concentrations of recombinant F7. (C) Cytokine secretion, including granzyme B, perforin, and IFN-γ in PBMCs co-cultured with HGC27 cells and treated with different concentrations of F7. (D) Flow cytometric analysis of $CD4^+$ T cells and regulatory T cells (Tregs; $CD4^+CD25^+FoxP3^+$) in MFC liver metastasis (LM) models. (E) Flow cytometric analysis of tumor cell apoptosis following co-culture with immune cells. (F) In vivo liver metastasis model showing that F7 knockdown reduced metastatic burden, whereas $CD8^+$ T-cell depletion partially restored liver metastasis. Representative hematoxylin and eosin (H&E) staining and quantification of the liver weight-to-body weight ratio are shown. (G) Immunofluorescence staining showing increased $CD8^+$ T-cell infiltration in sh-F7 liver metastatic lesions, which was reduced after $CD8^+$ T-cell depletion. (H) Flow cytometric analysis confirming $CD8^+$ T-cell depletion efficiency in vivo. *$p < 0.05$, **$p < 0.01$, ***$p < 0.001$, ****$p < 0.0001$.

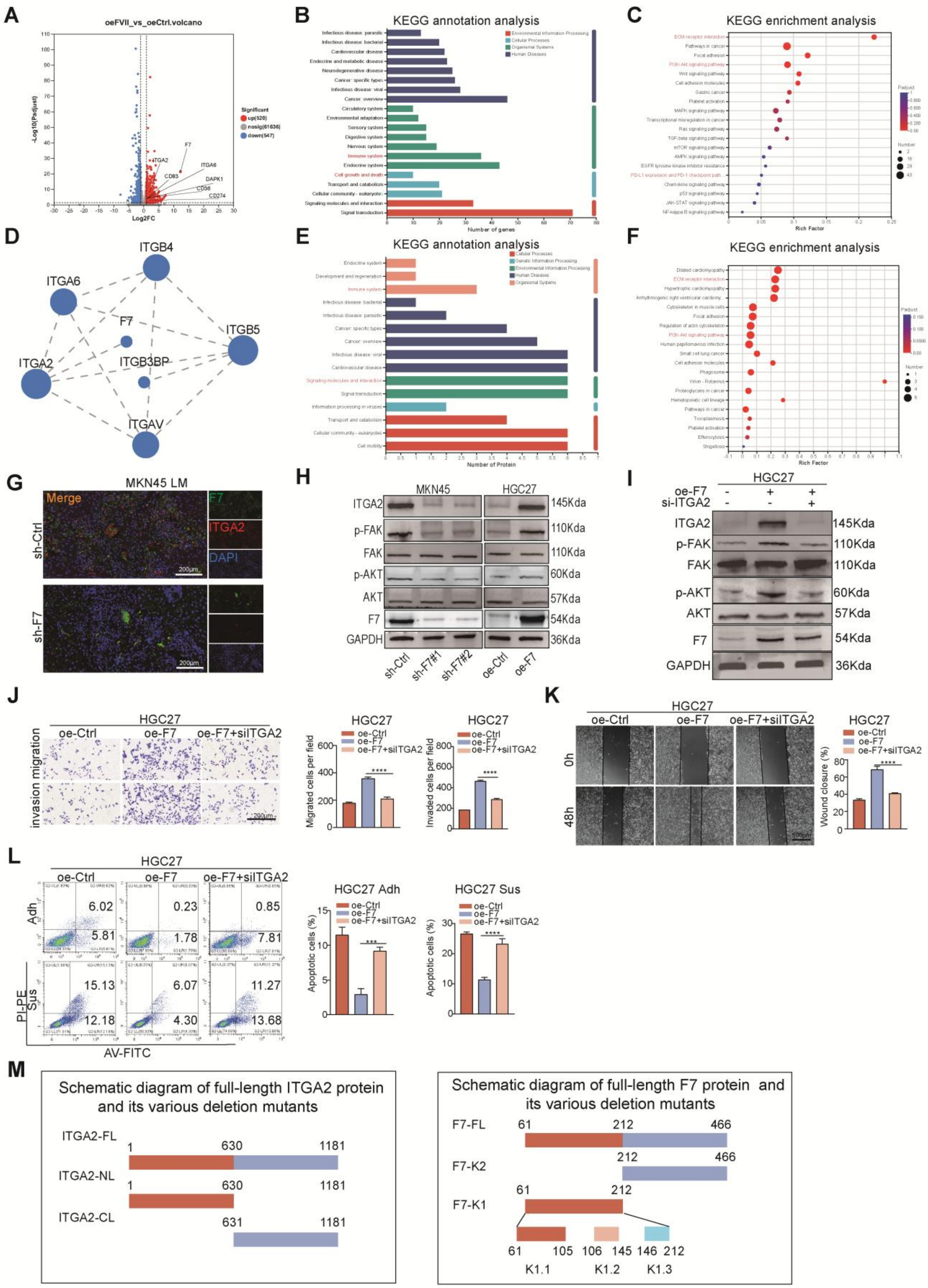
A
oeFVII_vs_oeCtrl.volcano
-Log10(Padjust)
Log2FC
Significant
up(520)
nosig(61636)
down(547)
F7
ITGA2
ITGA6
CD83
DAPK1
CD36
CD274
B
KEGG annotation analysis
Number of genes
C
KEGG enrichment analysis
Rich Factor
D
ITGB4
ITGA6
F7
ITGB5
ITGA2
ITGB3BP
ITGAV
E
KEGG annotation analysis
Number of Protein
F
KEGG enrichment analysis
Rich Factor
G
MKN45 LM
Merge
F7
ITGA2
DAPI
sh-Ctrl
sh-F7
200μm
H
MKN45
HGC27
ITGA2
p-FAK
FAK
p-AKT
AKT
F7
GAPDH
145Kda
110Kda
110Kda
60Kda
57Kda
54Kda
36Kda
sh-Ctrl
sh-F7#1
sh-F7#2
oe-Ctrl
oe-F7
I
HGC27
oe-F7
si-ITGA2
ITGA2
p-FAK
FAK
p-AKT
AKT
F7
GAPDH
145Kda
110Kda
110Kda
60Kda
57Kda
54Kda
36Kda
J
HGC27
oe-Ctrl
oe-F7
oe-F7+siITGA2
invasion migration
200μm
Migrated cells per field
Invaded cells per field
K
HGC27
oe-Ctrl
oe-F7
oe-F7+siITGA2
0h
48h
Wound closure (%)
L
HGC27
oe-Ctrl
oe-F7
oe-F7+siITGA2
Adh
Sus
PI-PE
AV-FITC
6.02
5.81
0.23
1.78
0.85
7.81
15.13
12.18
6.07
4.30
11.27
13.68
HGC27 Adh
HGC27 Sus
Apoptotic cells (%)
M
Schematic diagram of full-length ITGA2 protein and its various deletion mutants
ITGA2-FL
ITGA2-NL
ITGA2-CL
1
630
1181
631
Schematic diagram of full-length F7 protein and its various deletion mutants
F7-FL
F7-K2
F7-K1
61
212
466
105
106
145
146
K1.1
K1.2
K1.3

Figure S5. F7 promotes gastric cancer cell migration, invasion, and anoikis resistance through ITGA2-associated signaling. (A) RNA-seq volcano plot comparing F7-overexpressing cells with control cells (oe-F7 vs. oe-Ctrl), with differentially expressed genes highlighted. (B) KEGG annotation analysis of genes upregulated by F7 overexpression, showing enrichment of focal adhesion, ECM–receptor interaction, PI3K–AKT signaling, NF-κB-related pathways, and other top-ranked categories. (C) KEGG enrichment analysis of upregulated genes in oe-F7 cells, showing significant enrichment of ECM–receptor interaction, PI3K–AKT signaling, focal adhesion, and other cancer-related pathways. (D) Protein–protein interaction network showing predicted interactions between F7 and integrin family members, including ITGA2. (E) KEGG annotation analysis of F7-regulated proteins, showing enrichment of cancer-related, infection-related, and signaling pathway categories. (F) KEGG enrichment analysis of F7-regulated proteins, further showing enrichment of ECM–receptor interaction, focal adhesion, PI3K–AKT signaling, and other pathways. (G) Immunofluorescence staining showing the colocalization of F7 and ITGA2 in MKN45 liver metastatic lesions. F7 is shown in green, ITGA2 in red, and nuclei are counterstained with DAPI in blue. (H) Western blot analysis showing the effects of F7 knockdown or overexpression on ITGA2 expression and FAK and AKT phosphorylation in MKN45 and HGC27 cells. (I) Western blot analysis showing that ITGA2 knockdown attenuates F7-induced FAK and AKT phosphorylation in HGC27 cells. (J) Transwell migration and invasion assays showing that ITGA2 silencing suppresses F7-mediated enhancement of HGC27 cell migration and invasion. (K) Wound-healing assay showing that ITGA2 silencing attenuates F7-mediated enhancement of wound closure in HGC27 cells. (L) Flow cytometric analysis showing that ITGA2 knockdown partially reverses F7-mediated inhibition of apoptosis under adherent and suspension conditions. (M) Schematic diagrams of full-length and truncated ITGA2 and F7 constructs of ITGA2 and F7 used for domain-mapping assays. $*p < 0.05$, $**p < 0.01$, $***p < 0.001$, $****p < 0.0001$.

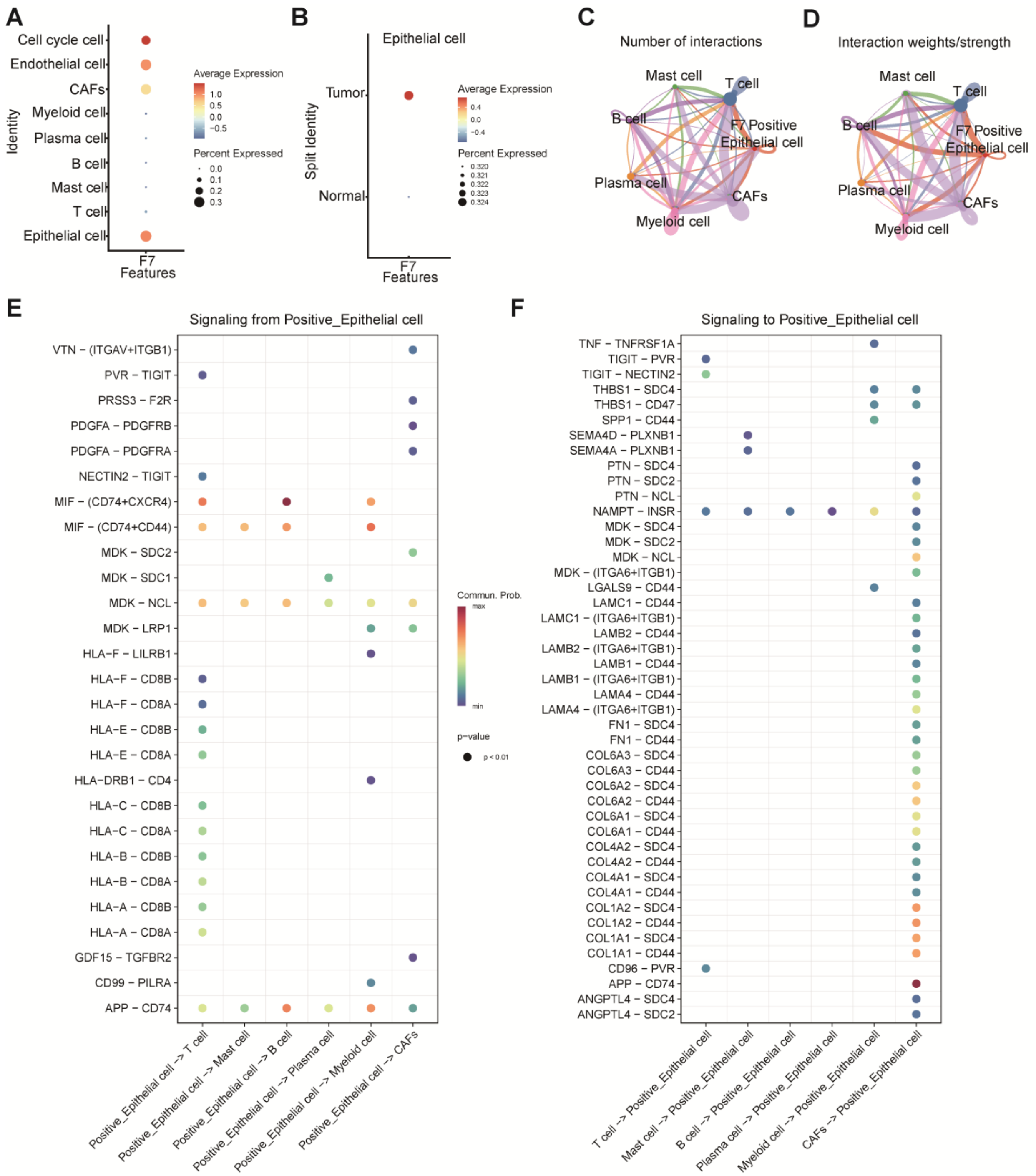

Figure S6. F7 expression and cell–cell communication patterns in the gastric cancer tumor microenvironment. (A) Heatmap showing the expression levels of F7 across different cell types. (B) Heatmap showing F7 expression in epithelial cells from tumor and normal tissues. (C) Network analysis showing the number of interactions between F7^{+} epithelial cells and immune or stromal cell populations. (D) Network analysis showing the interaction weights and strengths between F7^{+} epithelial cells and other cell types. (E) Outgoing signaling pathways from F7^{+} epithelial cells to other cell types. (F) Incoming signaling pathways from other cell types to F7^{+} epithelial cells.

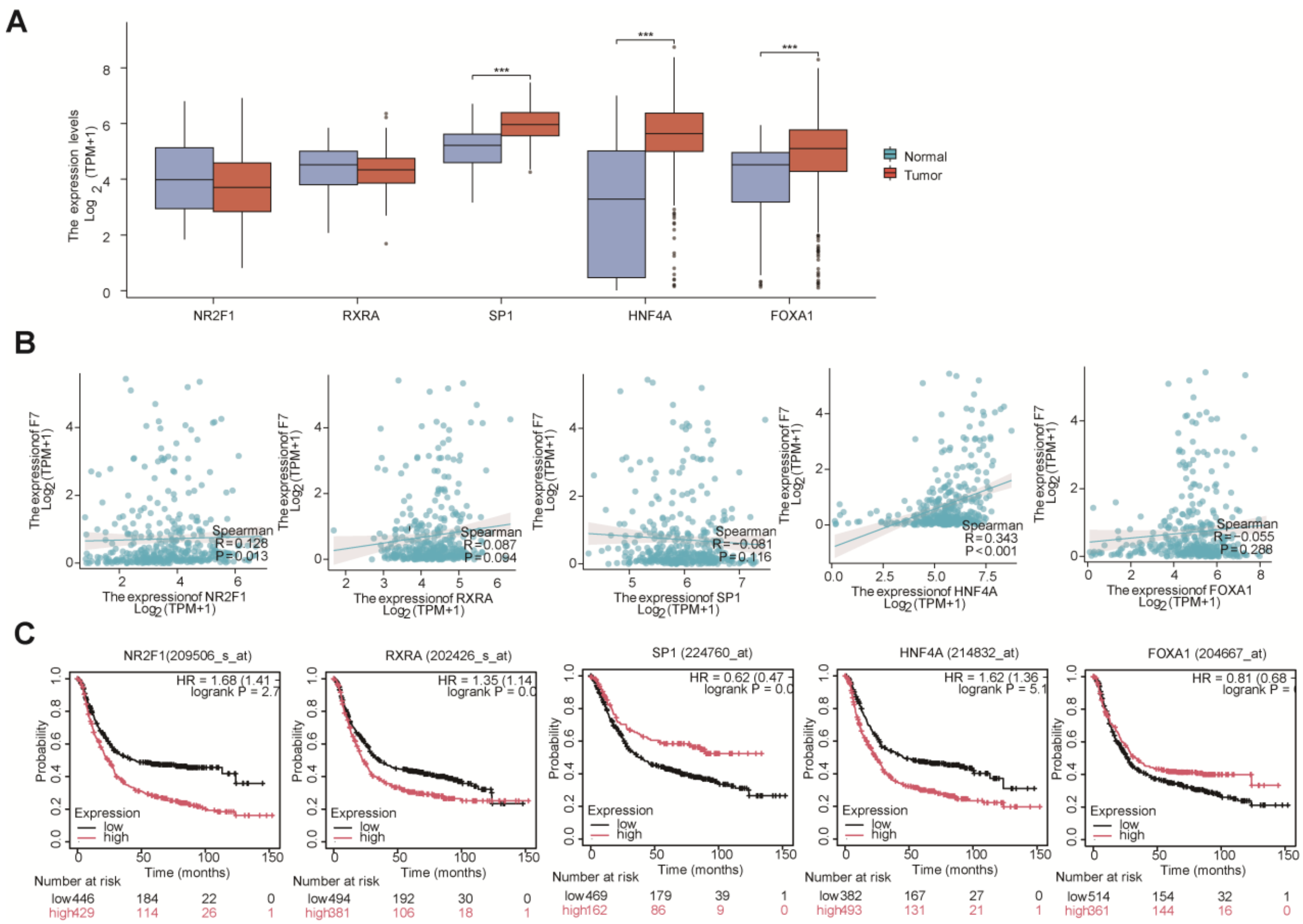

Figure S7. Expression patterns and clinical relevance of candidate transcription factors in gastric cancer. (A) Box plots comparing the expression levels of NR2F1, RXRA, SP1, HNF4A, and FOXA1 between normal tissues (blue) and tumor tissues (red). (B) Scatter plots showing correlations between F7 expression and the expression of selected transcription factors, including NR2F1, RXRA, SP1, HNF4A, and FOXA1, in tumor samples. Spearman's correlation coefficients (R) and *p* values are shown for each plot. (C) Kaplan–Meier survival curves for patients with gastric cancer stratified according to high or low expression of NR2F1, RXRA, SP1, HNF4A, and FOXA1. *$p < 0.05$, **$p < 0.01$, ***$p < 0.001$, ****$p < 0.0001$.

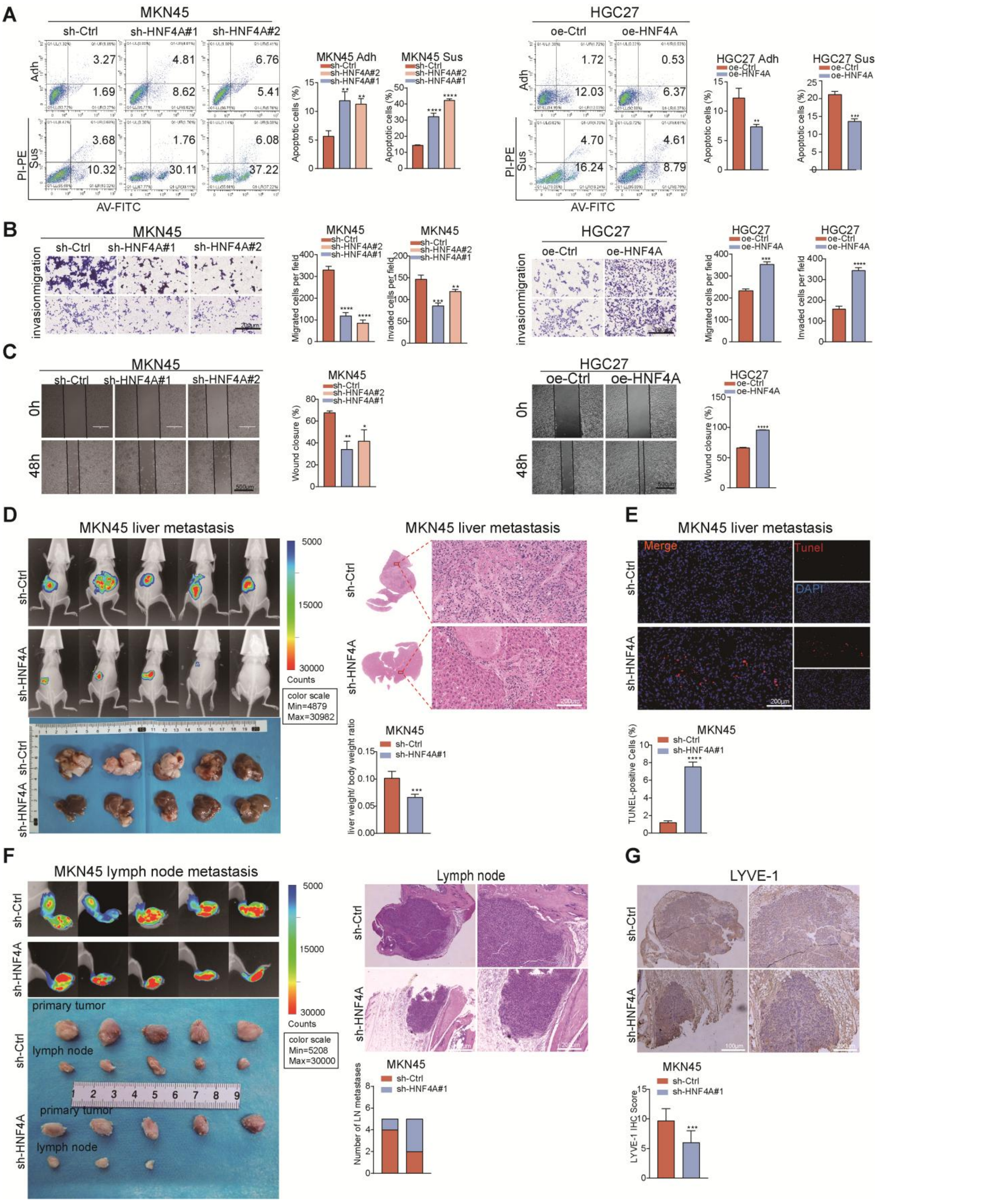

A
MKN45
sh-Ctrl
sh-HNF4A#1
sh-HNF4A#2
Adh
Sus
PI-PE
AV-FITC
3.27
4.81
6.76
1.69
8.62
5.41
3.68
1.76
6.08
10.32
30.11
37.22
MKN45 Adh
MKN45 Sus
Apoptotic cells (%)
HGC27
oe-Ctrl
oe-HNF4A
1.72
0.53
12.03
6.37
4.70
4.61
16.24
8.79
HGC27 Adh
HGC27 Sus
B
invasion
migration
Migrated cells per field
Invaded cells per field
C
0h
48h
Wound closure (%)
500μm
D
MKN45 liver metastasis
Counts
color scale
Min=4879
Max=30982
liver weight/ body weight ratio
E
Merge
Tunel
DAPI
TUNEL-positive Cells (%)
F
MKN45 lymph node metastasis
primary tumor
lymph node
color scale
Min=5208
Max=30000
Lymph node
Number of LN metastases
G
LYVE-1
LYVE-1 IHC Score

Figure S8. HNF4A promotes gastric cancer cell survival, migration, and metastasis. (A-C) Flow cytometry, Transwell invasion, and wound-healing assays show that HNF4A knockdown increases apoptosis and reduces migration and invasion in MKN45 cells, whereas HNF4A overexpression promoted cell survival, migration, and invasion in HGC27 cells under adherent (Adh) and suspension (Sus) conditions. (D-E) Liver metastasis model showing that HNF4A knockdown reduced metastatic burden and increased tumor cell apoptosis, as demonstrated by bioluminescence imaging, gross liver images, hematoxylin and eosin (H&E) staining, and TUNEL assays. (F-G) Lymph node metastasis analysis showing that HNF4A knockdown reduced lymph node metastasis and lymphangiogenesis, as demonstrated by bioluminescence imaging (BLI), H&E staining, and LYVE-1 immunohistochemical (IHC) staining. *$p < 0.05$, **$p < 0.01$, ***$p < 0.001$, ****$p < 0.0001$.

‘

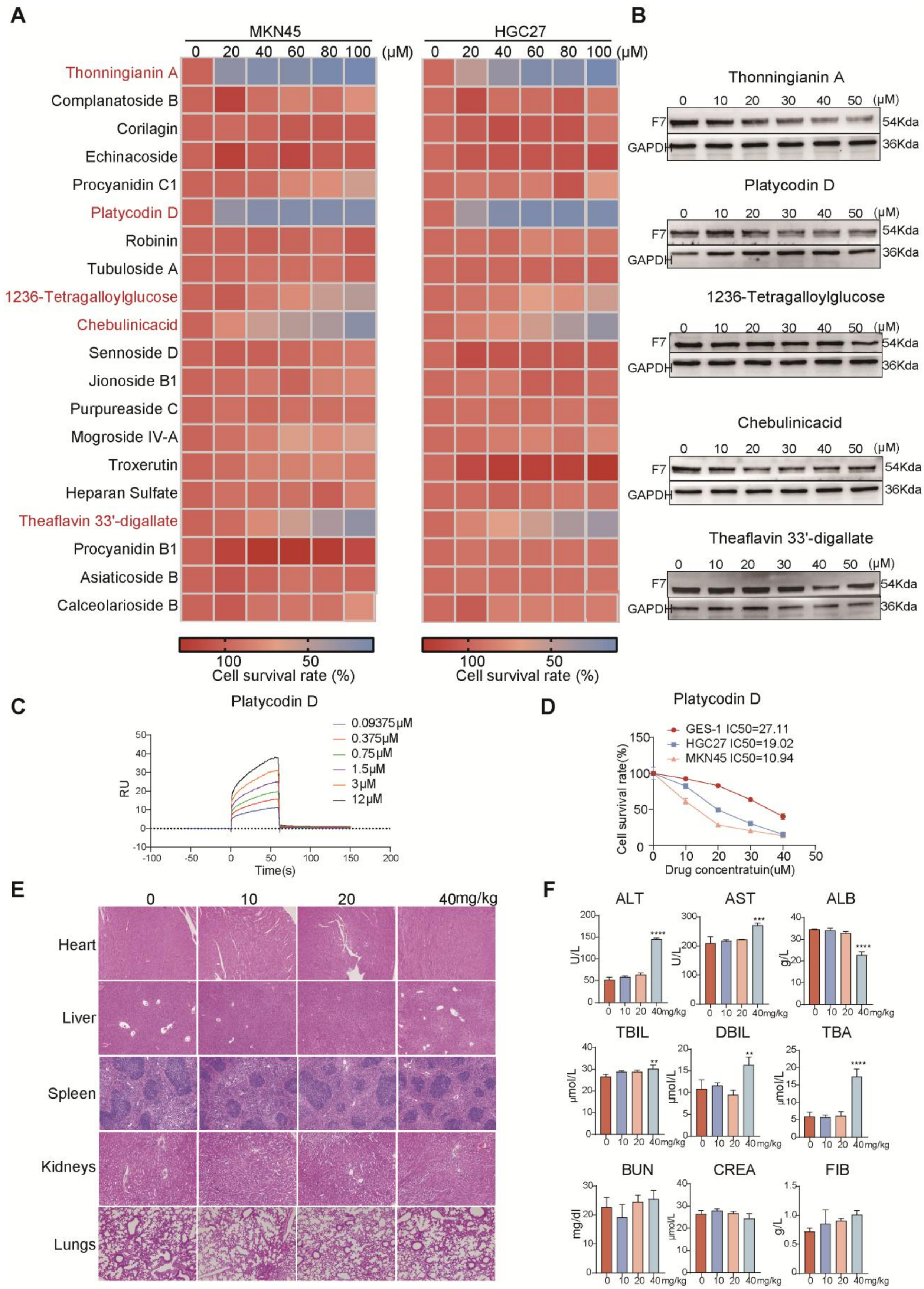
A
MKN45
0 20 40 60 80 100 (μM)
HGC27
0 20 40 60 80 100 (μM)
Thonningianin A
Complanatoside B
Corilagin
Echinacoside
Procyanidin C1
Platycodin D
Robinin
Tubuloside A
1236-Tetragalloylglucose
Chebulinicacid
Sennoside D
Jionoside B1
Purpureaside C
Mogroside IV-A
Troxerutin
Heparan Sulfate
Theaflavin 33'-digallate
Procyanidin B1
Asiaticoside B
Calceolarioside B
100 50
Cell survival rate (%)
B
Thonningianin A
0 10 20 30 40 50 (μM)
F7 54Kda
GAPDH 36Kda
Platycodin D
1236-Tetragalloylglucose
Chebulinicacid
Theaflavin 33'-digallate
C
Platycodin D
0.09375μM
0.375μM
0.75μM
1.5μM
3μM
12μM
RU
Time(s)
D
Platycodin D
GES-1 IC50=27.11
HGC27 IC50=19.02
MKN45 IC50=10.94
Cell survival rate(%)
Drug concentratuin(uM)
E
0 10 20 40mg/kg
Heart
Liver
Spleen
Kidneys
Lungs
F
ALT
AST
ALB
TBIL
DBIL
TBA
BUN
CREA
FIB
U/L
g/L
μmol/L
mg/dl
0 10 20 40mg/kg

Figure S9. Screening of small-molecule inhibitors targeting F7. (A) Heatmap showing cell survival rates in MKN45 and HGC27 cells treated with different compounds. The color gradient represents cell survival, with red indicating higher survival and blue indicating lower survival. (B) Western blot analysis showing F7 protein expression in MKN45 cells treated with increasing concentrations of Thonningianin A, Platycodin D, 1,2,3,6-tetragalloylglucose, chebulinic acid, and theaflavin 3,3′-digallate. (C) Surface plasmon resonance (SPR) assay showing the binding affinity of Platycodin D to F7 at concentrations ranging from 0.06–12 μM. (D) Cell survival assay showing dose–response curves for Platycodin D treatment in GES-1, HGC27, and MKN45 cells. (E) Histological analysis of major organs, including the heart, liver, spleen, kidneys, and lungs, from mice treated with Thonningianin A at doses of 0, 10, 20, and 40 mg/kg. (F) Blood biochemistry analysis showing the levels of ALT, AST, ALB, TBIL, DBIL, TBA, BUN, CREA, and FIB in mice treated with Thonningianin A at doses of 0, 10, 20, and 40 mg/kg. *$p < 0.05$, **$p < 0.01$, ***$p < 0.001$, ****$p < 0.0001$.

Tables S1 Primers and siRNA used in this study

Primiers	Sequences
F7	F:ACCCACAGTTGAATATCCATGT<br>R:CCTCCAGTTCTTGATTTTGTCG
HNF4A	F:AGGAGCTCCCTCAAGGATGA<br>R:GATGGGGACGTGTCTGGTG
ITGA2	F:CACAAAGACACAGGTGGGGT<br>R:TGGGATGTCTGGGATGTTGC
GAPDH	F:AGAAGGCTGGGGCTCATTTG<br>R:AGGGGCCATCCACAGTCTTC
Site1	F:TGCTCTGCTCTCTGGACTGA<br>R:GGGAGGTGATTCCTTCCCAA
Site2	F:GAGGCCATGAGCCACTGAAT<br>R:GAGTAGATGGCTGGCTGGTC
Site3	F:CTGCACGCTGGCAACAAA<br>R:AAGGGTGACAGAGGGATGG
Site4	F:GACAGCAGGTAGAAATGACG<br>R:TGGGATTACAGGCGTGAG
Site5	F:GGGTCACCTGACCTTTCTCC<br>R:CACAGGCGTCCTCTGAACAC
si-ITGA2	Sense:GGUGCCUGCAGAAGAAUAUTT<br>Antisense:AUAUUCUUCUGCAGGCACCTT

Tables S2 Antibodies used in this study

Name	Supplier/Cat no	Apply
F7	Proteintech/23058-1-AP	WB 1:1000 IHC/IF1:200
HNF4A	Abcam/EPR19265-130	WB 1:3000 IHC/IF 1:200
ITGA2	Proteintech/30703-1-AP	WB 1:2000 IHC/IF1:200
FAK	Proteintech/12636-1-AP	WB 1:1000
p-FAK	Proteintech/83933-1-RR	WB 1:1000
NF-κB	Proteintech/80979-1-RR	WB 1:1000
P-NF-κB	Proteintech/82335-1-RR	WB 1:1000 IHC/IF1:200

AKT	Proteintech/10176-2-AP	WB 1:1000
P-AKT	Proteintech/66444-1-Ig	WB 1:1000
PD-L1	Invitrogen/14-5983-82	WB 1:1000
GAPDH	Proteintech/60004-1-Ig	WB 1:10000
β -actin	Proteintech/66009-1-Ig	WB 1:10000
CD3	MAB-0740	IHC
CD4	RMA-1086	IHC
CD8	MAB-1031	IHC
CD68	MAB-0863	IHC
CD206	Proteintech/18704-1-AP	IHC 1:500
CD8 m	Abcam/EPR20305	IF 1:500
CollagenI	Servicebio/GB11022-3	IF 1:200
GranzymeB	Proteintech/13588-1-AP	IF 1:200
CD3	MULTISCIENCES/F11003A01	FlowC
CD4	MULTISCIENCES/F11004A04	FlowC
CD8	MULTISCIENCES/F11008A06	FlowC
CD3-Fitcm	BioLegend/100203	FlowC
CD4-PerCP-Cy5.5m	BioLegend/100539	FlowC
CD8-PE/Cyanine7m	BioLegend/100721	FlowC
GranzymeB-PEm	BioLegend/396405	FlowC
CD25-APC m	BioLegend/102011	FlowC
FOXP3-PEm	BioLegend/118903	FlowC
PD-L1-PE	Invitrogen/MA5-60238	FlowC

Table S3. Predicted interaction residues between F7 and ITGA2 based on molecular docking analysis

Receptor	Ligand	Hydrogen bond Interaction	Electrostatic interaction
F7	ITGA2	ILE150-GLY793	GLU192-ARG597
		PRO134-THR667	HIS317-GLU544
		LYS221-GLU547	GLU137-HIS786
			ASP146-LYS665
			ASP316-LYS559
			HIS144-GLU664

Tables S4 Candidate small molecule inhibitors

**Name**	**CAS**	**docking score**	**Mw**	**Formula**
1,2,3,6-Tetragalloylglucose	79886-50-3	-15.985	788.57	$C_{34}H_{28}O_{22}$
Tubuloside A	112516-05-9	-14.783	828.76	$C_{37}H_{48}O_{21}$
Thonningianin A	271579-11-4	-14.681	874.71	$C_{42}H_{34}O_{21}$

Echinacoside	82854-37-3	-14.033	786.73	C35H46O20
Chebulinic acid	18942-26-2	-13.856	956.68	C41H32O27
Sennoside D	37271-17-3	-13.718	848.76	C42H40O19
Complanatoside B	142473-99-2	-13.511	756.66	C33H40O20
Jionoside B1	120406-37-3	-13.308	814.7809	C37H50O20
Purpureaside C	108648-07-3	-13.223	786.73	C35H46O20
Platycodin D	58479-68-8	-12.782	1225.32	C57H92O28
Mogroside IV-A	88901-41-1	-12.598	1125.29	C54H92O24
Troxerutin	7085-55-4	-12.177	742.68	C33H42O19
Heparan Sulfate	9050-30-0	-12.109	1201.01	C12H19NO20S3 (monomer)
Theaflavin 3,3'-digallate	30462-35-2	-12.018	868.70	C43H32O20
Procyanidin B1	20315-25-7	-11.872	578.52	C30H26O12
Corilagin	23094-69-1	-11.864	634.45	C27H22O18
Asiaticoside B	125265-68-1	-11.861	975.12	C48H78O20
Procyanidin C1	37064-30-5	-11.720	866.77	C45H38O18
Calceolarioside B	105471-98-5	-11.674	478.45	C23H26O11
Robinin	301-19-9	-11.405	740.66	C33H40O19
